# Fricke gel dosimeter with improved sensitivity for low‐dose‐level measurements

**DOI:** 10.1120/jacmp.v17i4.5626

**Published:** 2016-07-08

**Authors:** Mauro Vaiente, Wladimir Molina, Lila Carrizales Silva, Rodolfo Figueroa, Francisco Malano, Pedro Pérez, Mauricio Santibañez, José Vedelago

**Affiliations:** ^1^ Institute of Physics E. Gaviola—CONICET Cordoba Argentina; ^2^ University of Cordoba Cordoba Argentina; ^3^ Departamento de Ciencias Físicas Universidad de La Frontera Temuco Chile; ^4^ Instituto Venezolano de Investigaciones Científicas Caracas Venezuela; ^5^ 1.3 Departamento de Física, FCEFQyN Universidad Nacional de Río Cuarto Río Cuarto Argentina

**Keywords:** radiation detection, Fricke gel dosimetry, radiology

## Abstract

Fricke solution has a wide range of applications as radiation detector and dosimetry. It is particularly appreciated in terms of relevant comparative advantages, like tissue‐equivalence when prepared in aqueous media like gel matrix, continuous mapping capability, independence of dose rate and incident direction, as well as linear dose response. This work presents the development and characterization of an improved Fricke gel system, based on modified chemical compositions, making possible its application in clinical radiology due to its improved sensitivity. Properties of standard Fricke gel dosimeter for high‐dose levels are used as a starting point, and suitable chemical modifications are introduced and carefully investigated in order to attain high resolution for low‐dose ranges, like those corresponding to radiology interventions. The developed Fricke gel radiation dosimeter system achieves the expected typical dose‐dependency, showing linear response in the dose range from 20 up to 4000 mGy. Systematic investigations including several chemical compositions are carried out in order to obtain an adequate dosimeter response for low‐dose levels. A suitable composition from among those studied is selected as a good candidate for low‐dose‐level radiation dosimetry consisting of a modified Fricke solution fixed to a gel matrix containing benzoic acid along with sulfuric acid, ferrous sulfate, Xylenol orange, and tridistilled water. Dosimeter samples are prepared in standard vials for in‐phantom irradiation and further characterization by spectrophotometry measuring visible light transmission and absorbance before and after irradiation. Samples are irradiated using typical X‐ray tubes for radiology and calibrated Farmer‐type ionization chamber is used as reference to measure dose rates inside phantoms at vial locations. Once sensitive material composition is optimized, dose‐response curves show significant improvement regarding overall sensitivity for low dose levels. The aim of this work consists of implementing the optimized gel dosimeter to perform direct measurements of absorbed dose in samples irradiated during microcomputed tomography scanning in order to preliminary assess dose levels for further scanning of small animals for further applications in veterinary and paleontology. As a first attempt, dose distributions were measured in water‐equivalent phantoms having dimensions comparable to small animals, 100 to 1000 cm^3^, approximately. According to the obtained results, it is found that the proposed method shows satisfactory reliability and adequate performance for a promising gel dosimetry system.

PACS number(s): 87.53.Bn, 87.57.uq, 87.59.‐e

## I. INTRODUCTION

Ionizing radiations are crucial to the diagnosis and treatment of diseases due to their ability to interact and transmit through human tissues, thus motivating worldwide increase in applications of ionizing radiation‐based techniques. An increase in problems associated with the determination of the absorbed dose in the different clinical applications has arisen as a direct result of growth in use of ionizing radiation in medicine and veterinary. The mechanisms for estimating the dose in clinical practices involving ionizing radiations have become critical. There is currently a need to develop and deploy new dosimetry techniques to cover a broad range of techniques for both diagnostic and therapy, which represents a demanding challenge.

The development of chemical dosimeters suitable for different demanding applications in quality assurance and beam characterization has a large history.^1^, ^2^ The ferrous sulfate (Fricke) solution had been well used for radiation dosimetry for decades. One of the most important issues about this development relates to the stabilization of spatial/geometric distribution of absorbed dose by incorporating the aqueous Fricke solution into a gelatin matrix. Further addition of Xylenol orange (XO) marker modified light absorption properties, shifting the absorption peak from 302 nm for standard Fricke gel (FG) to 580 nm, approximately. The resulting system is known as XO‐added Fricke gel (FXG).

It is noteworthy that dosimetry systems based on FXG have significant advantages: high lighted tissue equivalence, beam quality independence (*a priori*, at least to conventional techniques (i.e., not hadronic)), regardless of both quasi‐linear energy transfer^3^, ^4^, ^5^, ^6^, ^7^ and dose rate.^3^, ^5^, ^8^, ^9^ Although diffusion of ferric ions represents a nonnegligible drawback,^1^, ^2^, ^3^, ^10^ suitable preparation/manipulation protocols along with prompt analysis provided to FXG the capability to obtain three‐dimensional dosimetry mappings. Besides, this requires moderate cost of implementation and operation when using light transmission imaging as readout method.^3^, ^11^, ^12^, ^13^, ^14^

One of the main scopes of chemical dosimetry was to develop dosimeters with improved sensitivity to be used for measurements of low dose levels. It is well known that the performance of chemical dosimeters is strongly dependent upon the chemical yield known as G‐value and, of course, it is limited by instrument detection limit. Some decades ago, Dr. Gupta's group proposed a dosimetry system that incorporated benzoic acid to the traditional FXG, obtaining promising performance for low‐dose‐level measurements.^(9)^ It was found that the presence of benzoic acid increases the G‐value for ferric ions $\left(G({\text{Fe}}^{3+})\right)$, presenting an appreciable change in visible light absorption around 540 nm, depending on gelatin matrix. Ionizing radiation produces radicals in ferrous‐Xylenol solution, forming complexes of ferric ions with the XO marker. Determination of stable free radicals produced during irradiation can be achieved using different analytical methods, like magnetic resonance or an inexpensive alternative readout based on spectrophotometry, proposed by Gupta et al.^15^, ^16^ The FXG dosimetry by thin layers optically analyzed, originally developed by Prof. Gambarini,^17^, ^18^ has been demonstrated to be a suitable and reliable tool for wide applications, including standard radiation therapy modalities, like intensity‐modulated radiotherapy,^(19)^ measurements of dose profiles and multiple fields irradiations,^11^, ^20^, ^21^ and high‐dose‐rate brachytherapy,^(22)^ among other conventional and nonconventional procedures. The latter include mainly Boron neutron capture therapy (BNCT), for which this technique proved to be even capable of assessing the different dose contributions, then separating therapeutic from other components.^23^, ^24^, ^25^, ^26^, ^27^, ^28^, ^29^

The present study proposes the development of a ferrous‐Xylenol Fricke gel dosimeter with dose sensitivity characteristics that can perform absorbed‐dose measurements for radiological applications. The aim of this work is to develop, characterize, and execute preliminary implementation of a dosimetric radiation detector with high enough sensitivity capable of performing reliable absorbed‐dose distribution measurements for low‐dose level, as required by radiological procedures.^3^, ^30^ The study begins with a pre‐established composition of FXG for the measurement of radiation dose levels typically used for therapeutic purposes (i.e., relative high doses).^3^, ^11^, ^18^, ^19^ Although this formulation is not used for further measurements, its good performance for dosimetry at high dose levels supports its use as a starting point for this study.

It is investigated whenever dose‐response sensitivity can be improved by suitable modifications to chemical composition and changes during our elaboration process, according to information available in the literature about the effect of benzoic acid in FXG dosimeters. Investigations are carried out by modifying the system dedicated to relative high doses by incorporating chemical components, like benzoic acid^3^, ^9^, ^31^, ^32^, ^33^ in its preparation. Comparisons between the developed FXG system with conventional dosimetry systems are accomplished with the aim of validating it as a reliable dosimetry system. Irradiation experiences are performed according to typical setups of computed tomographic (CT) practices. Once developed, the dosimetry system is intended to be used for characterization of absorbed doses by small animals exposed to radiological procedures by microcomputed tomography (μCT) scanning.

## II. MATERIALS AND METHODS

### A. Fricke gel preparation and characterization methods

The development of a new dosimeter was carried out introducing modifications to the initial reference consisting of a FXG dedicated to the high radiation dose levels estimation^(3,18,19)^composed of a solution of 124.38 mM of porcine skin gel 300 Bloom, 0.6 mM of ferrous ammonium sulfate (Mohr's salt), 28 mM of sulfuric acid, 2 mM of Xylenol orange, and 96% of tri‐distilled water. The study about dose response of benzoic Fricke gel was initiated using the composition reported originally by Gupta et al.:^9^, ^15^ 0.2 mM ferrous ammonium sulfate, 0.05 mM sulfuric acid, 0.2 mM Xylenol orange, 5 mM benzoic acid. The chemical composition of sensitive material was modified in the first instance, using suggestions provided in literature, mainly by Gupta et al.^15^, ^30^, ^34^, ^35^ Also, suggestions by other authors were taken into account regarding the preparation process,^3^, ^6^ as well as cooling and storage procedures.^(3,6,13)^The formulation of benzoic Fricke gel is going to be used in the present study as the base for characterization of dosimetry performance of the μCT facility.

Dosimeter samples were elaborated in similar conditions to FXG without benzoic acid.^(3,6,22)^The preparation was poured into standard spectrophotometric plastic vials ($10\times 10\times 40\;{\text{mm}}^{3}$). Cuvettes' caps, previously prepared with parafilm to avoid external exchange, were placed immediately, closing samples almost hermetically. Then, gel dosimeter cuvettes were stored at 4°C for 24 hrs before irradiation. In fact, it was found that this procedure, being relatively similar to that commonly followed for Fricke dosimetry without benzoic acid, was suitable for attaining acceptable performance, as reported.

**Figure 1:**
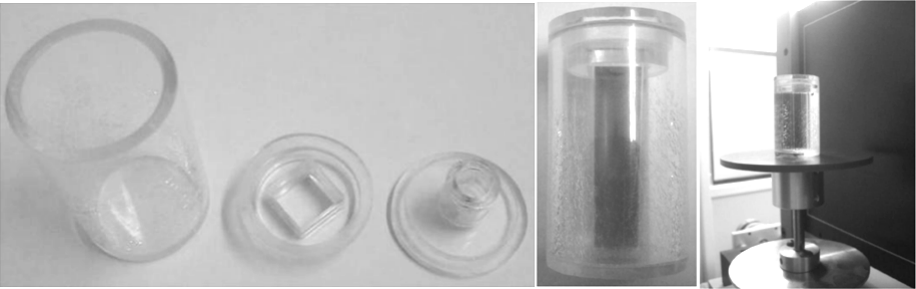
Cylindrical phantom (left) with FXG vial (middle) for μCT setup (right).

For the new design, the procedure consisted of the controlled variation of one component of the solution at a time, while keeping remaining components unchanged. In this way, although the results were obtained for the dosimeter output characteristics with respect to these variations, they proceeded to attain the most favorable outcome in view of achieving increments in both sensitivity and stable behavior at the time of measurement (well defined linearity with least possible deviation margin between measurements). The chemical composition resulted from variations in the following ranges: benzoic acid between 2.5–10 mM, sulfuric acid between 15–40 mM, ferrous ammonium sulfate from 0.2–0.5 mM, and XO between 0.1–0.3 mM. Characterization was performed by considering the relationship between the difference in absorbance of nonirradiated and irradiated samples with water reference using X‐rays. Samples of FXG were placed inside the cylindrical phantom for their irradiation, as shown in Fig. 1.

Any system that is to be considered a suitable dosimeter has to fulfill acceptable characteristics of reproducibility and independence with the radiation beam quality.^4^, ^6^, ^33^, ^36^, ^37^ These were tested to judge if the new solution is applicable in low‐dose‐level dosimetry. Reproducibility, being one of the most relevant properties of the dosimeter, was studied using experiments aimed to test whether the new solution could reproduce tolerable dose dependence response. The minimum values of sensitivity and linearity of the dose dependence were assessed as variables in all cases.

Dependence with respect to the characteristics of the radiation beam quality was also investigated. The experiments conducted with the above purpose were devoted to evaluate the independence of the new solution with respect to the characteristics of the radiation beam. The X‐ray tube electron current was fixed at 40 mA and radiation quality was varied by changing accelerating voltage (20, 25, 45, and 50 kVp), thus getting beams with different half‐value layer (HVL). Measurements were performed for 125 and 250 mGy dose values at dosimeter sample position, checked by ionization chamber.

Preliminary studies characterizing optical properties showed that the presence of benzoic acid in the composition produced variations in optical absorbance. The change in the absorption peak moved from almost 580 nm (for FXG dosimeters without benzoic acid) to around 540–550 nm. Furthermore, several studies were performed to select the most convenient wavelength range for reading out optical density differences. Dose‐response curve showed different effects due to the postirradiation elapsed time when reading out optical density differences (ΔOD) in different wavelength ranges. The overall more stable situation corresponded to 20 nm range centered at 547 or 551 nm, performing measurements spaced by 1 nm. Then, the wavelength range 540 to 560 nm was established as the most convenient way for assessing ΔOD to be correlated with absorbed dose. It was also verified that correlation in dose response did not vary significantly when performing measurements spaced by 10 nm; as a result, this modality was adopted. Specific and deep investigations dedicated to the analysis of absorbance changes at different wavelengths represent a valuable tool for characterizing effects of different species present in the solutions. However, this particular issue does not constitute a goal of this work.

Finally, specific experiments were carried out in order to establish preliminary the overall performance of the developed system in clinical‐like situations. Tomographic images were made configuring the X‐ray tube to 50 kVp, 5 mA, and using Zirconium filtering of 0.5 mm and extra aluminum filtering of 1 mm at the exit window of beam. This filtering process ensured high‐definition images and little detector saturation. The setup of FXG samples in the tomographic system is presented in Fig. 1.

The method consisted of measuring absorbance differences with respect to a reference sample of distilled water inside a quartz vial. Samples of FXG were measured before and after irradiation to establish ΔOD in correspondence to absorbed dose. This procedure was used to assess the dose‐response curve. After irradiation, FXG samples were kept at 4°C for 30 min. Then, they were placed at room temperature that was controlled to be stable (within 1°C) during the experiment. Readout was performed 10 min later. This process is useful to reduce the effect of thermal variations in the FXG dosimeters.^(36)^ The calibration curve should match the linear regression that predicts the response of the dependent variable with respect to changes in the independent variable. Therefore, it had to be adjusted to regression model, with the dependent variable corresponding to the variation of the optical density differences (ΔOD) — calculated as the ratio of logarithms of transmittance before and after irradiation — of the irradiated with respect to the nonirradiated sample using the absorbed dose as independent variable. Then, operational transformations of ΔOD provided the corresponding values of unknown absorbed dose.

Finally, comparisons were performed between readouts by benzoic Fricke gel dosimeters and ionization chamber. For the measurements with Fricke gel dosimeters, it was considered that, due to the symmetry of CT scanning, they might be acceptable to associate them to homogeneous dose distribution. Thus, meaning that readouts corresponded to almost point dose values representing the mean effective absorbed dose in sensitive volume. Dose rate for 1 min of irradiation was determined, then, measuring the processing time of CT, the total dose during the whole imaging process was estimated.

### B. Materials and instrumentation for radiology

A microcomputed tomography facility developed in laboratory provided with dedicated mechanical and software‐controlled electronic systems was used to irradiate dosimeter samples positioned in sample holders. In addition to mechanics and electronics, the CT facility incorporates detection equipment using a flat‐panel Varian model PaxScan 2020+ (Varian Medical Systems, Palo Alto, CA) which allows energy dynamic range of 20 keV to 150 keV.

A continuous emission X‐ray tube was used as a radiation source with electron current intensity within 5–50 mA, voltage in the range 20–60 kV, provided with different available anode materials. Adaptations to the X‐ray sources, mechanics/electronics devices, radiation detection systems, and dedicated postprocessing software are the main constituents of the CT facility.

**Figure 2:**
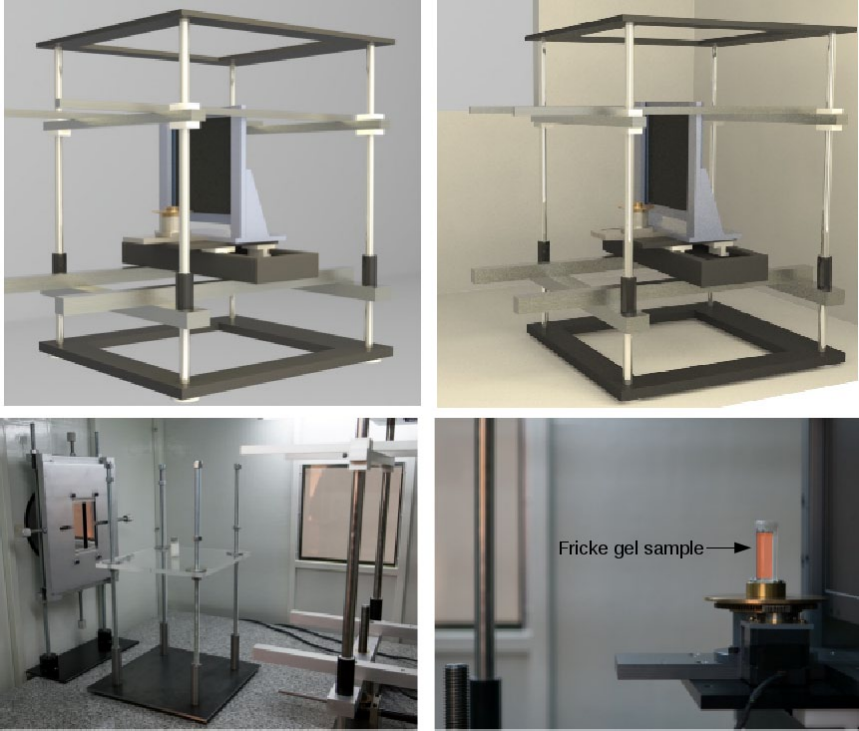
Design (computer‐aided design CAD) of bench top cone‐beam μCT (top left and right) and pictures of typical μCT setup during sample scanning (bottom left and right).

It is optimized for high spatial resolution, which makes it a micro‐CT (μCT). Both digital radiography and tomography imaging may be performed at the facility, shown in Fig. 2.

The μCT setup consists of the photon beam from the conventional X‐ray source, a rotation sample holder, and the detection system. Dedicated electromechanics and software synchronize and control the scanning process, as well as further computed tomography reconstruction and visualization by means of conical‐beam CT reconstruction algorithms, according to the μCT setup.

A spectrophotometer 1205 Vis from UNICO (Dayton, NJ) was used for measuring both absorbance and transmittance of the samples covering a spectrum within a wide dynamic range from infrared up to ultraviolet (325–1000 nm), reproducibility of (±1 nm), a transmittance range from 0% to 125%, and an absorbance photometric accuracy of 0.004 in optical density (OD).

Samples of tri‐distilled water in UV quartz container with tolerance of 1% corresponding to 220 nm were used as the reference standard for the measurement of absorbance difference. FXG samples were prepared using standard spectrophotometric acrylic containers. An ionization chamber PTW‐Freiburg TN 30013 (Freiburg, Germany) with certified calibration factors (megavoltage and kilovoltage photon beams) in water was used as conventional reference dosimetry system for calibration and comparisons.

A cylindrical phantom (30 mm diameter, 50 mm height, and 2 mm thick acrylic walls) was constructed for tomographic applications. Additionally, special adaptations were designed (see Fig. 2) that were devoted to estimating the dose at center of phantom. The adapters ensure that both FXG and ionization chamber remain properly positioned inside the phantom so that centers of both sensitive volumes are in the same position.

## III. RESULTS

### A. Elaboration of Fricke gel with improved sensitivity

**Figure 3:**
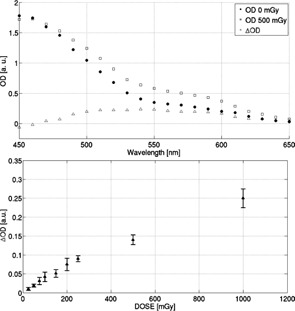
Optical density spectra before and after sample irradiation along with corresponding ΔOD delivering 500 mGy (top) and dose response (bottom) reporting typical dependence of recorded ΔOD on absorbed dose for a standard and benzoic FXG dosimeters. Linear regressions are calculated for the intervals (250, 20000) mGy and (20, 1000) mGy, respectively.

As mentioned, dose response was characterized in terms of variations in optical properties. Optical density differences between before and after sample irradiation were correlated with absorbed dose, as shown in Fig. 3. As reported, optical properties represent a suitable option to evaluate absorbed dose. As seen in Fig. 3, it was decided to use a narrow (±10 nm) wavelength range centered at the absorption peak (550 nm, approximately) for calculation of ΔOD.

The formulation containing benzoic acid shows a remarkable sensitivity increase. Assuming, *a priori*, an overall linear trend, linear regression was calculated for dose responses, obtaining the mean values for the corresponding slopes —for the FXG:

(1) $$\Delta {\text{OD}}_{\text{FXG}}=(1.43\pm 0.08)10^{-5}D[\text{mGy}]‐0{.0065(R}^{2}=0.996)$$

and for the benzoic (BFXG):

(2) $$\Delta {\text{OD}}_{\text{BFXG}}=(2.39\pm 0.11)10^{-4}D[\text{mGy}]‐0{.012(R}^{2}=0.992)$$

The first step was to study the effect of changing the acidity (pH) level of the standard (high‐dose‐level optimized) material. Solutions prepared with 2.5 mM and 10 mM of sulfuric acid showed a dramatic increase in optical density even under protection (avoiding light exposure and controlling temperature below 15°C), even with just 5 min elapsed. In both cases, the resulting values for light transmission around 550 nm were almost indistinguishable from zero‐scale, thus avoiding determination of light absorbance due to saturation effects. Therefore, it evidences a high degree of chemical instability, which generated spontaneous oxidation. In fact, the solutions were oxidized after 25 min, making them unusable for subsequent applications. The addition of 5 mM of benzoic acid provided stability compared to previous solutions, since 5 min after elaborated, the preparation showed no visible changes in its optical density. It remained stable for up to 30 min of observation after processing in conditions of shelter, even without assessing the sensitivity of this composition. This empirical observation, though sensitivity had not yet been quantitatively evaluated, suggested that this concentration of benzoic acid appeared to be convenient for the preparation of the FXG solution. Further experiences supported this assumption.^9^, ^37^, ^38^

**Figure 4:**
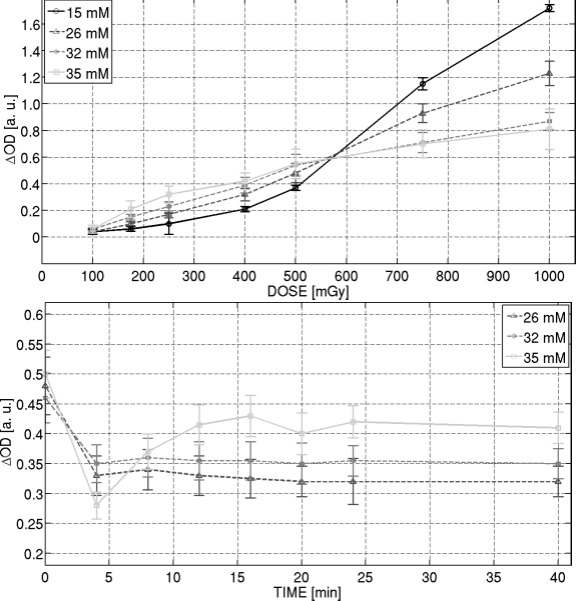
Dose response of FXG with different concentrations of sulfuric acid (top) and corresponding short/long‐term readout stability (bottom) delivering 750 mGy.

With respect to the variation between 15 and 35 mM of sulfuric acid in the dose level range (<500 mGy), it was found that the increase in the solution was proportionally related to the increase in the obtained sensitivity, as summarized in Fig. 4.

Considering the priority requirement that desired solution must satisfy the conditions of greater sensitivity with a good level of stability, a subsequent study was designed to evaluate the stability of the solution according to the time elapsed in measurement conditions. According to the results reported in Fig. 4, it can be seen that the solution prepared with 35 mM of sulfuric acid concentration was the most sensitive, but also the least stable. In view of the results presented in Fig. 4, it was observed that compositions with concentrations of sulfuric acid between 26 and 32 mM appeared as suitable choices.

**Figure 5:**
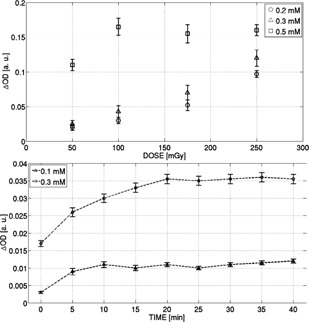
Dose response of FXG with different concentrations of FAS (top) and short/long‐term readout stability at different XO concentrations (bottom) delivering 750 and 250 mGy to the formulations, respectively.

Therefore, after analyzing the linear trend of the sensitivity response as a function of increasing the amount of sulfuric acid in the solution (as evidenced in Fig. 4), it was suitable to continue with further variations of component concentrations. The quantity of sulfuric acid was set to 29 mM, which is the representative midpoint. It was found that 29 mM sulfuric acid concentration presents the best postirradiation stability when compared with the other concentrations, as reported in Fig. 4. Although not here reported, similar behavior as reported in Fig. 4 was observed for the range of sulfuric acid concentrations investigated (15–40 mM). After preliminary examination, the results obtained from varying the concentration of ferrous ammonium sulfate (FAS) practically showed increased sensitivity for greater amounts of FAS available to interact and react with the incident radiation, as shown in Fig. 5. However, it was observed that saturation happens at lower doses when the amount of FAS is increased. As a consequence, although the sensitivity might be improved, the corresponding dose range of validity for the linearity would be significantly reduced.

The composition corresponding to 0.3 mM of FAS (keeping concentration of other components to: 29 mM sulfuric acid, 0.1 mM XO, 5 mM benzoic acid dissolved in a matrix consisting of 124.38 mM gelatin and 96% of water) showed sufficient enough sensitivity to differentiate low‐dose values as 50 mGy, and it was also verified that this formulation attains acceptable stability because the ΔOD mean value remained almost constant after 20 min. Conversely, the solution with 0.2 mM of ferrous ammonium sulfate was not able to differentiate dose values lower than 175 mGy. Finally, it was found that preparation with the concentration of 0.5 mM FAS did not exhibit linear response within an acceptably wide enough dose range. Therefore, the concentration of 0.3 mM of FAS was used to continue the elaboration process to improve sensitivity for low dose levels.

Regarding the variation of XO, the obtained results showed that this component is closely related to the stability of the system. Stability studies regarding elapsed time postirradiation suggest that the composition prepared with 0.1 mM XO is more stable, as reported in Fig. 5. Although both XO concentrations 0.1 mM and 0.3 mM exhibit acceptable stability after 20 min, it has to be mentioned that long‐term stability (10 hrs) shows better performance for 0.1 mM concentration of XO, as reported in Fig. 5.

Finally, the composition corresponding to the higher sensitivity for the purposes of this study consists of 5 mM benzoic acid, 29 mM sulfuric acid, 0.3 mM ferrous ammonium sulfate, and 0.1 mM XO. All components were suspended in a gel matrix gel 124.38 mM porcine skin gel (300 Bloom) to 96% of volume with tri‐distilled water.

**Figure 6:**
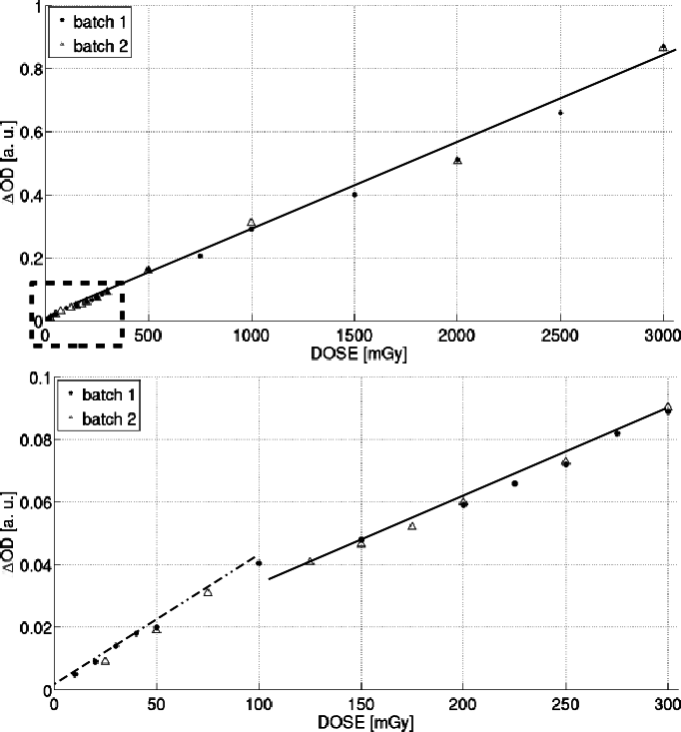
Dose response of FXG along with reproducibility for different elaboration batches (top) and zoom for the region indicated by rectangle (bottom). Linear regression parameters are: $(4.12\pm 0.08)\times 10^{-4}$ and $R^{2}=0.997$ for the dose range 20–100 mGy and $(3.79\pm 0.15)\times 10^{-4}$ and $R^{2}=0.992$ for the dose range 100–3000 mGy.

The output (dose‐response curves) obtained through the change in optical density with respect to the absorbed dose showed a linear behavior with two well‐defined slopes, as seen in Fig. 6. It was possible to achieve high enough resolution for dose levels even lower than 20 mGy that were clearly distinguishable from background.

This behavior is due to changes in the nature of the solution due to the exhaustion of any of its components. In fact, the sensitivity tests indicate that the component that mainly affects this behavior is the ferrous ammonium sulfate. Incorporating processing protocols and process improvements achieved sufficient sensitivity to distinguish dose levels below 20 mGy, maintaining linear behavior with a double slope at a point 100 mGy and similar consistency for all preparations.

### B. Characterization of the developed high‐sensitivity Fricke gel dosimeter

Three different elaborations of the optimized composition were evaluated, changing absorbed dose ranges during irradiation. Sample positioning in phantom and further readout were maintained, hence ensuring that the obtained results could be further compared in order to investigate if the output remained stable, regardless of the method of characterization.

Two different batches of high‐sensitivity Fricke solution were prepared separately. The first batch was evaluated for the range of 10–3000 mGy, whereas the second one was irradiated with doses in the interval 25–3000 mGy. As presented in Fig. 6, the same behavior of linearity was observed between solutions regardless of characterization method.

Table 1 reports measurements of average absorbed doses corresponding to different beam qualities according to applied accelerating voltages.

The results reported in Table 1 support that this dosimetry system response seems to be independent of beam quality, due to statistically indistinguishable results. The independence of beam quality could be also evaluated in terms of sensitivity of the system (characterized by the slope of linear regression) for the different accelerating voltages.

This preliminary evidence suggests that the developed sensitive dosimeter is practically independent of beam quality, as least in radiological applications.

**Table 1:** Dose from dosimeter response (A OD) for different radiological beam qualities

*Accelerating Voltage (kVp)*	*Reference Delivered Dose 125 mGy*	*Reference Delivered Dose 250 mGy*
20	0.14±0.05	0.27±0.06
25	0.13±0.07	0.25±0.06
45	0.14±0.07	0.24±0.06
50	0.15±0.06	0.26±0.07

### C. Implementation of the dosimeter for radiology

**Figure 7:**
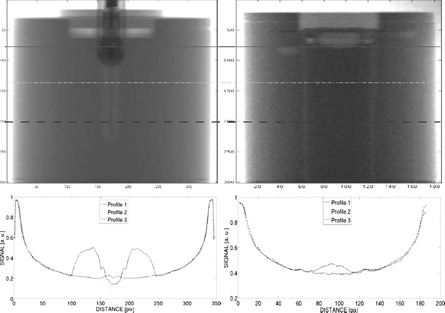
Absorption contrast from μCT of phantom containing ionization chamber (top left) and high‐sensitivity FXG (top right), along with profiles at different locations (bottom).

Tomography imaging of cylindrical phantom with inserted ionization chamber was performed in correspondence with Fig. 7. The reconstructed images showed an internal structure of the ionization chamber clearly differentiated from the water surrounding it. Ionization chamber sensitive (ionization) volume consists mainly of air and the electronics made of metallic pieces that produce changes in the X‐ray images when comparing ionization chamber and water. (The latter is conventionally considered as reference for human (soft) tissue (i.e., radiological equivalent to water.))

Conversely, in the case of the corresponding tomographic projection images obtained from FXG dosimeter presented in Fig. 7, it was confirmed that when placing the FXG dosimeter within a water‐equivalent medium, there are almost no differences in acquired signal. This suggests very similar radiation absorption and scattering properties between water and dosimeter. It indicates, therefore, good water equivalence, thus meaning (soft) tissue equivalence for clinical applications, as mentioned.

**Figure 8:**
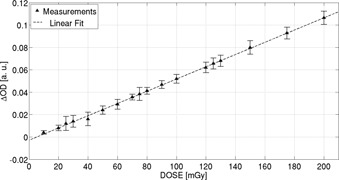
Calibration curve of high‐sensitivity FXG dosimeters used for μCT applications.

The calibration curve was obtained for the solution implemented in this test, getting absorbance sensitivity of $(48\pm 3)\times 10^{-4}/\text{mGy}$ and correlation coefficient $R^{2}=0.998$, as shown in Fig. 8. The obtained fit is valid and applies to the investigated dose range 25–200 mGy. Data reported in Fig. 8 correspond to mean values and associated standard deviation obtained from five samples performing independent measurements for each dose value.

**Figure 9:**
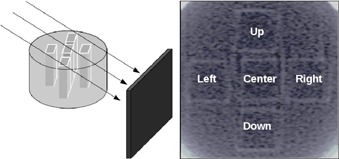
Sketch of μCT setup showing X‐ray beam, phantom with five FXG vials and detector (left) and reconstructed CT slice (right).

For dosimetric characterization of μCT irradiation five vials were placed inside a water‐equivalent phantom that was imaged in the μCT facility, as sketched in Fig. 9.

The object was imaged using a scanning modality of short acquisition times. The developed high‐sensitivity benzoic FXG dosimeters and ionization chamber were used to perform measurements during the μCT scanning process that lasted 12 min, using an intensity‐modulated beam. The obtained results are reported in Table 2.

The relative poor quality of the reconstructed slice, presented in Fig. 9, is due to the selected scanning modality with short irradiation times and reduced quantity of angular projection acquisition. This issue needs to be carefully investigated when performing μCT scanning, because high‐quality imaging may require excessively large exposures, thus not allowing its application for *in vivo* cases.

**Table 2:** Dose measurements (in mGy) during CT irradiation

*Dosimeter Location* (Fig. 9)	*Ionization Chamber*	*High Sensitivity Fricke Gel*
Center	134.1±0.1	118.0±3.1
Up	78.6±0.1	74.5±8.2
Down	100.6±0.2	94.2±6.4
Left	123.7±0.2	121.1±4.5
Right	100.0±0.1	102.0±2.1

## IV. DISCUSSION

First of all it should be emphasized that the overall performance of the developed dosimetric system exhibits typical characteristics of gel dosimeters, like linear response within dose range of interest,^1^, ^6^ reproducibility,^3^, ^6^, ^11^ tissue‐equivalence,^2^, ^3^, ^11^ and quite negligible photon beam energy dependence.^1^, ^2^, ^5^, ^8^, ^9^

Regarding preparation of FXG sensitive material, the approach of testing output response in correspondence to different chemical compositions, varying one component at a time, allowed to suitably improve dose response sensitivity. Once a high enough sensitive composition was achieved, further studies regarding stability were conducted in order to extend the available parameters for helping in composition selection.

Keeping controlled thermal history is recommended in order to avoid auto‐oxidation. The criterion of fixing storage time to 24 hrs for the Fricke solution with benzoic acid was established to this aim. The effect of storage time on dose response was previously checked; authors observed that longer storage times impact gel color. Moreover, excessively long elapsed time between gel elaboration and irradiation resulted in darker dosimeters (noticed with naked eye), thereby decreasing corresponding sensitivity. Before proceeding with experiments, the elapsed time between preparation and utilization that is required to arrive at chemical stability was carefully investigated. Also, Fricke solution preparation was always limited to batches not exceeding 500 ml filling standard vials (4 ml). Therefore, no large gel volumes were prepared for this work in order to avoid complications associated with long storage periods for reaching steady state conditions. In this sense, it should be addressed that caution is needed when analyzing whether results obtained with cuvettes might be applicable to dosimeters of larger volumes.

The dedicated optical analysis technique for the characterization of the high‐sensitivity FXG dosimeter demonstrated remarkable capability for assessing absorbed dose by means of transmission/absorption measurements. However, the implemented methodology, which appeared as optimal for analyzing homogeneous dose distribution within the sample, would need to be suitably modified if nonuniform dose distribution needed to be measured. In fact, one of the characteristics of the analyzing method that might be considered *a priori* as a limitation is that recorded dose values correspond to the integral along the optical path, thus providing an estimation of an “effective” dose value that obviously is not able to distinguish different doses along the integration path. That is why FXG samples should be prepared in “small enough” containers so that, when irradiated, it might be reasonable to consider that absorbed dose is constant within sample path.

Nevertheless, this potential drawback is suitably overcome by an improved 2D analyzing technique based on almost monochromatic sample transmission imaging.^3^, ^11^, ^18^, ^22^, ^24^, ^28^ As expected, the presence of linearity in dose response is reduced to specific dose ranges, observing initial dose interval without appreciable response, as well as saturation effects at higher dose levels. Moreover, the extension of this linearity range shows to be strongly correlated with linear slope, which represents the sensitivity of the system. The behavior at doses lower than linearity range corresponds to threshold effects exhibiting supralinear trend; whereas, for doses higher than linearity range, the output presents typical saturation effects, then sublinear trend, due to extinction of available reactive components.

It is expected that both sulfuric and benzoic acids concentrations should significantly influence the output, because the change in medium pH highly influences overall reactive grade. Similarly, concentration of FAS being the component providing the reactive agents (ferrous oxide) affects output. These results are in agreement with previous studies.^3^, ^4^, ^13^, ^37^ Sulfuric acid concentration needs to be carefully investigated. Its significant influence of dose response in hydrogels for dosimetry has been well reported.^4^, ^37^, ^38^ The amount of sulfuric acid used in this work was previously studied^(13)^ in order to avoid excessive reactivity, but the highest sensitivity possible was maintained. Additionally, sulfuric acid concentration was conditioned to ensure acceptable postirradiation stability, thus allowing flexibility for readout processes. As mentioned in previous works, it is clear that sulfuric acid used for the preparation of the Fricke dosimeter solution may contain trace impurities that can affect the yield of ferric ions. Small amounts of impurities, mainly organic, can affect the yield of ferric ions $G({\text{Fe}}^{3+})$. This issue is not accounted for during FXG preparation whenever the objective is limited to relative dosimetry, but it should be emphasized that it is required for absolute dosimetry. Regardless, it was not the intent of this study to exactly identify the cause of the effect and interested readers may refer to literature pertaining to this topic.^39^, ^40^ Thus, it was enough to assess adequate amount of sulfuric acid that satisfied sensitivity, reactivity, and stability requirements.

The effect of XO on dose response was carefully investigated during preliminary studies before the selection of adequate composition. Many authors reported that smaller changes in dose response correspond to changes in XO concentrations of 0.1 mM.^4^, ^13^, ^16^, ^37^, ^38^ The same was found with the preliminary results obtained for selecting dosimeter composition for this work. However, it was shown that different XO compositions, as may happen when using different suppliers — or even different lots from the same manufacturers — significantly affect dose responses.^(39)^ It was reported, in fact, that XO dye contains impurities, like cresol (red), acids (iminodiacetic, for example), and other not pure but semi‐XO compounds.^(13)^ Moreover, different XO compositions from different manufacturing processes may impact the chemical yield G value.^41^, ^42^, ^43^, ^44^ Preliminary tests for this work were conducted in order to characterize the performance of XO from different suppliers and lots (Anedra Research AG, Argentina lot 13445–11 and two flasks from Sigma‐Aldrich lots 52099A (St. Louis, MO). After examination, Sigma‐Aldrich XO was selected, because it exhibits higher sensitivity. Clearly, higher sensitivity is particularly critical when looking for low‐dose‐level dosimetry. Therefore, preliminary studies to establish the most convenient XO are a critical issue when looking for improved performance.

Regarding the performance of the dosimetric system for radiological applications, Fig. 7 presents dose response for the specific batch used for further measurements, which is needed for conversion to dose vials (used to measure the absorbed dose during X‐ray μCT scanning). In order to anticipate dose range, measurements with ionization chamber were performed before. This way it was possible to determine, *a priori*, that absorbed dose value should be lower than 100/120 mGy. Hence, the selected range (below 200 mGy) reported in Fig. 8 for obtaining calibration curve seemed to be a good choice. Values reported in Fig. 8 correspond to the average of five samples for each dose level. The selection of the optimal chemical composition for low‐dose‐level dosimetry was based on the compromise between high sensitivity (high slope values), extension of linearity range, and composition output stability, both after preparation and after irradiation. The obtained results for measuring low‐level absorbed dose in CT‐like setup, some of them summarized in Table 2, is evidence that the developed dosimetry system is capable of assessing similar dose estimations to ionization chamber, here used as an alternative for conventional systems.

A critical issue must be emphasized at this time: calibration curve provides information for dose conversion if necessary, but those samples for calibration must come from the same elaboration batch. The point in question is the importance of performing specific calibration for each FXG elaboration batch. For example, Fig. 8 reports dose‐response calibration for the specific batch that was further used for X‐ray μCT measurements, thus showing that each batch needs its own calibration. Moreover, it would be helpful to develop processes for batch‐to‐batch cross‐comparisons, which might be carefully implemented, attempting to integrate results from different elaboration batches.

Despite the noticeable overall agreement between high‐sensitivity FXG dosimeter and conventional method, ionization chamber and FXG dosimeter measurement volumes do not agree. Therefore, it should be expected that different dose readout values would be recorded if nonuniform irradiation is performed. Furthermore, as pointed out by results reported in Fig. 5, the presence of nonwater‐equivalent components of the ionization chamber might affect, even in a minor but potentially nonnegligible way, the radiation field, thus raising nondesired measurement alterations.

Regarding the dose‐response calibration of the batch dedicated to characterization of μCT facility, it must be pointed out that characterization of linear trend for low doses require further extensive investigations to get definitive characterization in terms of the different internal chemical processes affecting dose‐response curve. Therefore, dose was measured with ioniza‐tion chamber in the same conditions as gel samples, thus attempting to get the most reliable comparison. The presence of dose gradients during μCT scanning cannot be avoided, and there is no absolute guarantee that the dose gradients do, in fact, affect ionization chamber and gel dosimeters in an identical manner.

## V. CONCLUSIONS

The purpose of developing, characterizing, and preliminarily implementing a tissue‐equivalent high‐sensitivity FXG dosimeter was accomplished satisfactorily. It was possible to improve the sensitivity of the system, characterized by the slope in the dose‐response curve, obtaining a formulation 50 times, approximately, more sensitive than the standard one (see Fig. 3). Besides, it is important to underline that the present study does not intend to establish that benzoic acid would be indispensable for developing FXG with higher sensitivity.

It was demonstrated that novel FXG dosimetry system designed for low‐dose ranges certainly exhibits linear response capable of reliably measuring dose values within the range of 25–3000 mGy, observing readouts clearly distinguishable from the background and uncertainties less than 7% in all cases. Comparisons of gel dosimeters with ionization chamber suggest promising performance of the developed dosimeter for low‐dose levels.

The developed dosimetry system exhibits similarity to conventional system in the dose record, being indeed an indication that the developed system might constitute a promising tool as radiation detector potentially applicable to dosimetry in radiology and radiotherapy practices. The developed benzoic FXG was capable of performing reliable measurements of absorbed dose during μCT scanning. Although these measurements are preliminary carried out in water‐equivalent phantoms, the information collected is useful to predict the range of doses absorbed during μCT scanning of small animals. Next step involves the use of dedicated phantoms constructed according to animal morphology to insert benzoic Fricke gel dosimeters, avoiding the use of ionization chamber and associated limitations due to movements during μCT scanning.

## ACKNOWLEDGMENTS

This work was partially supported by project “Innovation Systems Research for Radiation Dosimetry: phase I (ISIDORA I) and phase II (ISIDORA II)” funded by University of Cordoba, Argentina and project ESPORA I (PIP 2014–2016 11220130100658CO) funded by CONICET, Argentina. This project was partially supported by DIFRO project DI16–6008 of Universidad de La Frontera.

## COPYRIGHT

This work is licensed under a Creative Commons Attribution 3.0 Unported License.

## Supporting information

Supplementary Material

Supplementary Material
