# Supplementary material for: Fricke gel dosimeter with improved sensitivity for low‐dose‐level measurements
Source: J Appl Clin Med Phys. 2016 Jul 8;17(4):402–17. doi: 10.1120/jacmp.v17i4.5626 (PMC5690050; doi:10.1120/jacmp.v17i4.5626)
Supplement: Supplementary file 1 — Supplementary Material [file ACM2-17-402-s001.doc]

Title:

Fricke gel dosimeter with improved sensitivity for low dose level measurements

**List all authors: first name, initial, last name:**

**Mauro Valente^1,2,3,*^ ; Wladimir Molina^4^ ; Lila Carrizales Silva^4^ ; Rodolfo Figueroa^3^ ; Francisco Malano^1,2,5^ ; Pedro Pérez^1,2,5^ ; Mauricio Santibañez^3^ ; José Vedelago^1,2,5^**

**Department, Institution, City, State, Country:**

*^1^ Institute of Physics E. Gaviola - CONICET, Cordoba, Argentina.*

*^2^ University of Cordoba, Cordoba, Argentina.*

*^3^ Departamento de Ciencias Físicas, Universidad de La Frontera, Temuco, Chile.*

*^4^ Instituto Venezolano de Investigaciones Científicas, Caracas, Venezuela.*

*^5^ University of Rio Cuarto, Cordoba, Argentina.*

^*^ Corresponding author: Prof. Mauro Valente, PhD.

Corresponding author’s full mailing address:

*Medina Allende esq. Haya de La Torre, Ciudad Universitaria*

*5000, Cordoba, ARGENTINA*

*Tel.: (+54) 3514334050 ext. 102
Fax: (+54) 3514334054*
Email: [valente@famaf.unc.edu.ar](mailto:valente@famaf.unc.edu.ar)

Submitted: February 11, 2015

Accepted: February 11, 2016

Running title: FGD improved for low dose level measurements

**Fricke gel dosimeter with improved sensitivity for low dose level measurements**

**Abstract**

Fricke solution has a wide range of applications as radiation detector and dosimetry. It is particularly appreciated in terms of relevant comparative advantages, like tissue-equivalence when prepared in aqueous media like gel matrix, continuous mapping capability, independence of dose rate and incident direction as well as linear dose response. This work presents the development and characterization of an improved Fricke gel system, based on modified chemical compositions making possible its application in clinical radiology due to its improved sensitivity. Properties of standard Fricke gel dosimeter for high dose levels are used as starting point and suitable chemical modifications are introduced and carefully investigated in order to attain high resolution for low dose ranges, like those corresponding to radiology interventions. The developed Fricke gel radiation dosimeter system achieves the expected typical dose-dependency, showing linear response in the dose range from 20 up to 4000 mGy. Systematic investigations including several chemical compositions are carried out in order to obtain an adequate dosimeter response for low dose levels. A suitable composition from among those studied is selected as a good candidate for low dose level radiation dosimetry consisting of a modified Fricke solution fixed to a gel matrix containing benzoic acid along with sulfuric acid, ferrous sulfate, Xylenol orange and tridistilled water. Dosimeter samples are prepared in standard vials for in-phantom irradiation and further characterization by spectrophotometry measuring visible light transmission and absorbance before and after irradiation. Samples are irradiated using typical X-ray tubes for radiology and calibrated Farmer type ionization chamber is used as reference to measure dose rates inside phantoms at vial locations. Once sensitive material composition is optimized, dose-response curves show significant improvement regarding overall sensitivity for low dose levels. The aim of this work consists on implementing the optimized gel dosimeter to perform direct measurements of absorbed dose in samples irradiated during micro computed tomography scanning in order to preliminary assessed dose levels for further scanning of small animals for further applications in veterinary and paleontology. As a first attempt, dose distributions were measured in water-equivalent phantoms having dimensions comparable to small animals, 100 to 1000 cm^3^, approximately. According to the obtained results, it is found that the proposed method shows satisfactory reliability and adequate performance for a promising gel dosimetry system.

***Keywords***: Radiation detection; Fricke gel dosimetry; Radiology

**1.- INTRODUCTION**

Ionizing radiations are crucial to the diagnosis and treatment of diseases due to their ability to interact and transmit through human tissues, thus motivating worldwide increase in applications of ionizing radiation-based techniques. An increase in problems associated with the determination of the absorbed dose in the different clinical applications has arisen as a direct result of growth in use of ionizing radiation in medicine and veterinary. The mechanisms for estimating the dose in clinical practices involving ionizing radiations have become critical. There is currently a need to develop and deploy new dosimetry techniques to cover a broad range of techniques for both diagnostic and therapy, which represents a demanding challenge.

The development of chemical dosimeters suitable for different demanding applications in quality assurance and beam characterization has a large history [Schreiner 2004, Baldock 2006]. The ferrous sulfate (Fricke) solution had been well used for radiation dosimetry for decades. One of the most important issues about this development regards the stabilization of spatial/geometric distribution of absorbed dose by incorporating the aqueous Fricke solution into a gelatin matrix. Further addition of Xylenol Orange (XO) marker modified light absorption properties, shifting the absorption peak from 302 nm for standard Fricke gel (FG) to 580 nm, approximately. The resulting system is known as XO-added Fricke gel (FXG).

It is noteworthy that dosimetry systems based on FXG have significant advantages: highlighted tissue equivalence, beam quality independence (a priori, at least to conventional techniques, *ie.* not hadronic), regardless of both quasi-linear energy transfer [Valente 2007, Svensson 1979, Sanguanmith 2011, Valente 2007b, Valente 2012] and dose rate [Gupta 1975 , Valente 2007, Sanguanmith 2011, Klassen 1999]. Although diffusion of ferric ions represents a non negligible drawback [Schreiner 2004, Baldock 2006, Valente 2007, Vedelago 2014], suitable preparation/manipulation protocols along with prompt analysis provided to FXG the capability to obtain three-dimensional dosimetry mappings requiring moderate cost of implementation and operation, when using light transmission imaging as readout method [Gambarini 2006, Valente 2007, Carrara 2007, Molina 2013, Gupta 1998].

One of the main scopes of chemical dosimetry was to develop dosimeters with improved sensitivity to be used for measurements of low dose levels. It is well known that the performance of chemical dosimeters is strongly dependent upon the chemical yield known as *G*-value and, of course, it is limited by instrument detection limit. Some decades ago, Dr. Gupta's group proposed a dosimetry system that incorporated benzoic acid to the traditional FXG obtaining promising performance for low dose level measurements [Gupta 1975]. It was found that the presence of benzoic acid increases the *G*-value for ferric ions (*G*(Fe^3+^)) presenting an appreciable change in visible light absorption around 540 nm, depending in gelatin matrix. Ionizing radiation produces radicals in ferrous-Xylenol solution forming complexes of ferric ions with the XO marker. Determination of stable free radicals produced during irradiation can be achieved using different analytical methods, like magnetic resonance or an inexpensive alternative readout based on spectrophotometry, proposed by Gupta et al. [Gupta 1985, Gupta 1973]. The FXG dosimetry by thin layers optically analyzed, originally developed by Prof. Gambarini [Gambarini 1994, Gambarini 2004], has been demonstrated to be a suitable and reliable tool for wide applications, including standard radiation therapy modalities, like intensity modulated radiotherapy [Tomatis 2007], measurements of dose profiles and multiple fields irradiations [Valente 2007 c, Gambarini 2006, Castellano 2007] and high dose rate brachytherapy [Carrara 2011], among others conventional and non-conventional procedures. The latter include mainly Boron Neutron Capture Therapy (BNCT), for which this technique proved to be even capable of assessing the different dose contributions, then separating therapeutic from other components [Gambarini 2010, Gambarini 2002, Gambarini 2004 b, Gambarini 1997, Bartesaghi 2009, Gambarini 2007, Gambarini 2014].

The present study proposes the development of a ferrous-Xylenol Fricke gel dosimeter with dose sensitivity characteristics that can perform absorbed dose measurements for radiological applications. The aim of this work is to develop, characterize and execute preliminary implementation of a dosimetric radiation detector with high enough sensitivity in order to be capable of performing reliable absorbed dose distribution measurements for low dose level as required by radiological procedures [Valente 2007, Gupta 1982]. The study begins with a pre-established composition of FXG for the measurement of radiation dose levels typically used for therapeutic purposes, *i.e.* relative high doses [Gambarini 2004, Tomatis 2007, Gambarini 2006, Valente 2007]. Although this formulation is not used for further measurements, its good performance for dosimetry at high dose levels supports its use as starting point for this study.

It is investigated whenever dose-response sensitivity can be improved by suitable modifications to chemical composition and changes during our elaboration process, according to information available in literature about the effect of benzoic acid in FXG dosimeters. Investigations are carried out by modifying the system dedicated to relative high doses by incorporating chemical components, like benzoic acid [Gupta 1975, Valente 2007, Wander 1968, Gore 1984, Kelly 1998] in its preparation. Comparisons between the developed FXG system with conventional dosimetry systems are accomplished with the aim of validating it as a reliable dosimetry system. Irradiation experiences are performed according to typical setups of computed tomographic (CT) practices. Once developed, the dosimetry system is intended to be used for characterization of absorbed doses by small animals exposed to radiological procedures by micro computed tomography (µCT) scanning.

**2.- MATERIALS AND METHODS**

**2.1.- Fricke gel preparation and characterization methods**

The development of a new dosimeter was carried out introducing modifications to the initial reference consisting of a FXG dedicated to the high radiation dose levels estimation [Gambarini 2004, Tomatis 2007, Valente 2007] composed of a solution of 124.38 mM of porcine skin gel 300 Bloom, 0.6 mM of ferrous ammonium sulfate (Mohr’s salt), 28 mM of sulfuric acid, 2 mM of Xylenol Orange and 96% of tri-distilled water. The study about dose-response of benzoic Fricke gel was initiated using the composition reported originally by Gupta et al. [Gupta 1985, Gupta 1975] 0.2 mM ferrous ammonium sulfate, 0.05 mM sulfuric acid, 0.2 mM Xylenol Orange, 5 mM benzoic acid. The chemical composition of sensitive material was modified in the first instance, using suggestions provided in literature, mainly by Gupta et al. [Gupta 1985, Gupta 1973 b, Gupta 1982, Upadhyay 1982]. Also, suggestions by other authors were taken into account regarding the preparation process [Valente 2007, Valente 2007 b] as well as cooling and storage procedures [Valente 2007, Valente 2007 b, Molina 2013]. The formulation of benzoic Fricke gel is going to be used in the present study as the base for characterization of dosimetry performance of the µCT facility.

Dosimeter samples were elaborated in similar conditions to FXG without benzoic acid [Carrara 2011, Valente 2007, Valente 2007 b]. The preparation was poured into standard spectrophotometric plastic vials (10×10×40 mm^3^). Cuvettes' caps, previously prepared with parafilm to avoid external exchange, were placed immediately closing samples almost hermetically. Then, gel dosimeter cuvettes were stored at 4 °C for 24 hours before irradiation. In fact, it was found that this procedure, being relatively similar to that commonly followed for Fricke dosimetry without benzoic acid, was suitable for attaining acceptable performance, as reported.

For the new design the procedure consisted of the controlled variation of one component of the solution at a time, while keeping remaining components unchanged. In this way, although the results were obtained for the dosimeter output characteristics with respect to these variations, they proceeded to attain the most favorable outcome in view of achieving increments in both sensitivity and stable behavior at the time of measurement (well defined linearity with least possible deviation margin between measurements). The chemical composition resulted from variations in the following ranges: benzoic acid between 2.5-10 mM, sulfuric acid between 15-40 mM, ferrous ammonium sulfate from 0.2-0.5 mM and XO between 0.1-0.3 mM. Characterization was performed by considering the relationship between the difference in absorbance of non-irradiated and irradiated samples with water reference using X-rays. Samples of FXG were placed inside the cylindrical phantom for their irradiation, as shown in Figure 1.


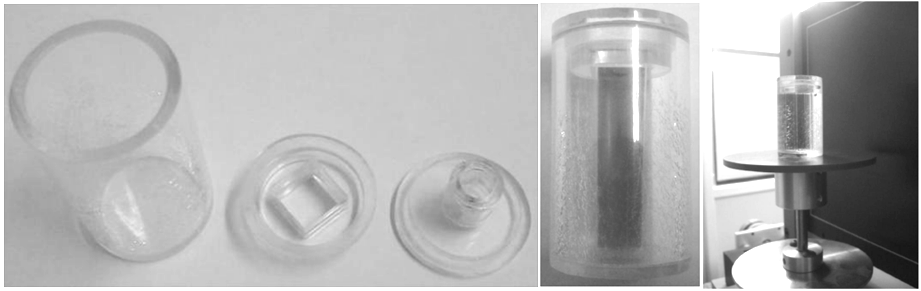


Figure 1.- Cylindrical phantom (left) with FXG vial (middle) for µCT setup (right).

Any system that is to be considered a suitable dosimeter has to fulfill acceptable characteristics of reproducibility and independence with the radiation beam quality [Kelly 1998, Valente 2007 b, Svensson 1979, Olding 2011, Dewhurst 1951]. These were tested to judge if the new solution is applicable in low dose level dosimetry. Reproducibility, being one of the most relevant properties of the dosimeter, was studied using experiments aimed to test whether the new solution could reproduce tolerable dose dependence response. The minimum values of sensitivity and linearity of the dose dependence were assessed as variables in all cases.

Dependence with respect to the characteristics of the radiation beam quality was also investigated. The experiments conducted with the above purpose were devoted to evaluate the independence of the new solution with respect to the characteristics of the radiation beam. The X-ray tube electron current was fixed at 40 mA and radiation quality was varied by changing accelerating voltage (20, 25, 45 and 50 kVp), thus getting beams with different half value layer (HVL). Measurements were performed for 125 and 250 mGy dose values at dosimeter sample position, checked by ionization chamber.

Preliminary studies characterizing optical properties showed that the presence of benzoic acid in the composition produced variations in optical absorbance. The change in the absorption peak moved from almost 580 nm (for FXG dosimeters without benzoic acid) to around 540-550 nm. Furthermore, several studies were performed to select the most convenient wavelength range for reading out optical density differences. Dose-response curve showed different effects due to the post-irradiation elapsed time when reading out optical density differences (Δ *OD*) in different wavelength ranges. The overall more stable situation corresponded to 20 nm range centered at 547 or 551 nm, performing measurements spaced by 1 nm. Then, the wavelength range 540 to 560 nm was established as the most convenient way for assessing Δ *OD* to be correlated with absorbed dose. It was also verified that correlation in dose-response did not vary significantly when performing measurements spaced by 10 nm, then it was adopted this modality. Specific and deep investigations dedicated to the analysis of absorbance changes at different wavelengths represent a valuable tool for characterizing effects of different species present in the solutions. However, this particular issue does not constitute a goal of this work.

Finally, specific experiments were carried out in order to establish preliminary the overall performance of the developed system in clinical-like situations. Tomographic images were made configuring the X-ray tube to 50 kVp, 5 mA and using Zirconium filtering of 0.5 mm and extra aluminum filtering of 1 mm at the exit window of beam. This filtering process ensured high definition images and little detector saturation. The setup of FXG samples in the tomographic system is presented in Figure 1.

The method consisted of measuring absorbance differences with respect to a reference sample of distilled water inside a quartz vial. Samples of FXG were measured before and after irradiation to establish Δ *OD* in correspondence to absorbed dose. This procedure was used to assess the dose-response curve. After irradiation, FXG samples were kept at 4 °C during 30 minutes. Then, they were placed at room temperature that was controlled to be stable (within 1 °C) during the experiment. Readout was performed 10 minutes later. This process is useful to reduce the effect of thermal variations in the FXG dosimeters [36]. The calibration curve should match the linear regression that predicts the response of the dependent variable with respect to changes in the independent variable. Therefore, it had to be adjusted to regression model, with the dependent variable corresponding to the variation of the optical density differences (Δ *OD*); calculated as the ratio of logarithms of transmittance before and after irradiation; of the irradiated with respect to the non-irradiated sample using the absorbed dose as independent variable. Then, operational transformations of Δ *OD* provided the corresponding values of unknown absorbed dose.

Finally, comparisons were performed between read outs by benzoic Fricke gel dosimeters and ionization chamber. For the measurements with Fricke gel dosimeters, it was considered that, due to the symmetry of CT scanning, they might be acceptable to associate them to homogeneous dose distribution. Thus, meaning that readouts corresponded to almost point dose values representing the mean effective absorbed dose in sensitive volume. Dose rate for one minute of irradiation was determined, then, measuring the processing time of CT, the total dose during the whole imaging process was estimated.

**2.2.- Materials and Instrumentation for radiology**

A micro computed tomography facility developed in laboratory provided with dedicated mechanical and software-controlled electronic systems was used to irradiate dosimeter samples positioned in sample holders. In addition to mechanics and electronics, the CT facility incorporates detection equipment using a flat panel Varian model PaxScan 2020+ which allows energy dynamic range of 20 keV to 150 keV.

A continuous emission X-ray tube was used as a radiation source with electron current intensity within 5-50 mA, voltage in the range 20-60 kV, provided with different available anode materials. Adaptations to the X-ray sources, mechanics-electronics devices, radiation detection systems and dedicated post-processing software are the main constituents of the CT facility.

It is optimized for high spatial resolution, which makes it a micro-CT (µCT). Both digital radiography and tomography imaging may be performed at the facility, shown in Figure 2.

The µCT setup consists of the photon beam from the conventional X-ray source, a rotation sample holder and the detection system. Dedicated electro-mechanics and software synchronize and control the scanning process as well as further computed tomography reconstruction and visualization by means of conical beam CT reconstruction algorithms; according to the µCT setup.

A spectrophotometer 1205 Vis from UNICO was used for measuring both absorbance and transmittance of the samples covering a spectrum within a wide dynamic range from infrared up to ultraviolet (325-1000 nm), reproducibility of (±1 nm), a transmittance range from 0% to 125% and an absorbance photometric accuracy of 0.004 in optical density (*OD*).


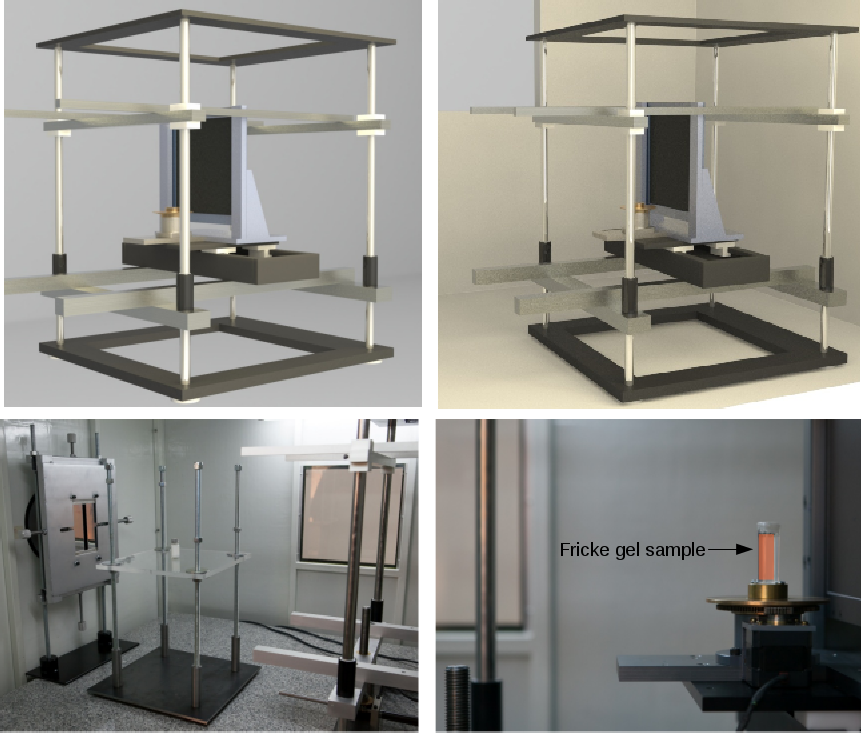


Figure 2.- Design (computer-aided design - CAD) of bench-top cone beam µCT (top left and right) and pictures of typical µCT setup during sample scanning (bottom left and right).

Samples of tri-distilled water in UV quartz container with tolerance of 1% corresponding to 220 nm were used as the reference standard for the measurement of absorbance difference. FXG samples were prepared using standard spectrophotometric acrylic containers. An ionization chamber PTW-Freiburg TN 30013 with certified calibration factors (megavoltage and kilovoltage photon beams) in water was used as conventional reference dosimetry system for calibration and comparisons.

A cylindrical phantom (30 mm diameter, 50 mm height and 2 mm thick acrylic walls) was constructed for tomographic applications. Additionally, special adaptations were designed (see Figure 2) that were devoted to estimating the dose at center of phantom. The adapters ensure that both FXG and ionization chamber remain properly positioned inside the phantom so that centers of both sensitive volumes are in the same position.

**3.- RESULTS**

**3.1.- Elaboration of Fricke gel with improved sensitivity**

As mentioned, dose response was characterized in terms of variations in optical properties. Optical density differences between before and after sample irradiation were correlated with absorbed dose, as shown in Figure 3. As reported, optical properties represent a suitable option to evaluate absorbed dose. According to Figure 3, it was decided to use a narrow (±10 nm) wavelength range centered at the absorption peak (550 nm, approximately) for calculation of Δ*OD*.


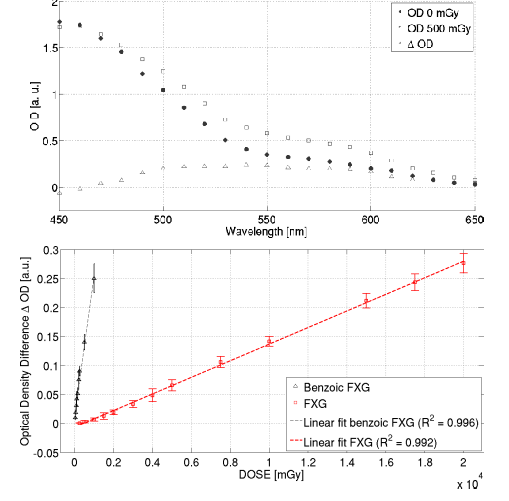


Figure 3.- Optical density spectra before and after sample irradiation along with corresponding Δ*OD* delivering 500 mGy (top) and dose-response (bottom) reporting typical dependence of recorded Δ*OD* on absorbed dose for a standard and benzoic FXG dosimeters. Linear regressions are calculated for the intervals [250, 20000] mGy and [20, 1000] mGy; respectively.

The formulation containing benzoic acid shows a remarkable sensitivity increase. Assuming, *a priori*, an overall linear trend, linear regression was calculated for dose-responses, obtaining the mean values for the corresponding slopes: for the FXG: **Δ*OD_FXG_*** *= (1. 43±0.08) 10^-5^* ***D*** *[mGy] – 0.0065 (R^2^=0.996)* and for the benzoic (BFXG): **Δ*OD_BFXG_*** *= (2. 39±0.11) 10^-4^* ***D*** *[mGy] – 0.012 (R^2^=0.992).*

The first step was to study the effect of changing the acidity (pH) level of the standard (high dose level optimized) material. Solutions prepared with 2.5 mM and 10 mM of sulfuric acid showed a dramatic increase in optical density even under protection (avoiding light exposure and controlling temperature below 15°C), even with just 5 minutes elapsed. In both cases, the resulting values for light transmission around 550 nm were almost indistinguishable from zero-scale, thus avoiding determination of light absorbance due to saturation effects. Therefore, it evidences a high degree of chemical instability, which generated spontaneous oxidation. In fact, the solutions were oxidized after 25 minutes, making them unusable for subsequent applications. The addition of 5 mM of benzoic acid provided stability compared to previous solutions, since five minutes after elaborated, the preparation showed no visible changes in its optical density. It remained stable for up to 30 minutes of observation after processing in conditions of shelter, even without assessing the sensitivity of this composition. This empirical observation, though sensitivity had not yet been quantitatively evaluated, suggested that this concentration of benzoic acid appeared to be convenient for the preparation of the FXG solution. Further experiences supported this assumption [Gupta 1975, Dewhurst 1951, Palm 2000].

With respect to the variation between 15 and 35 mM of sulfuric acid in the dose level range (< 500 mGy), it was found that the increase in the solution was proportionally related to the increase in the obtained sensitivity, as summarized in Figure 4.

Considering the priority requirement that desired solution must satisfy the conditions of greater sensitivity with a good level of stability, a subsequent study was designed to evaluate the stability of the solution according to the time elapsed in measurement conditions. According to the results reported in Figure 4, it can appreciated that the solution prepared with 35 mM of sulfuric acid concentration was the most sensitive but also the least stable. In view of the results presented in Figure 4, it was observed that compositions with concentrations of sulfuric acid between 26 and 32 mM appeared as suitable choices.


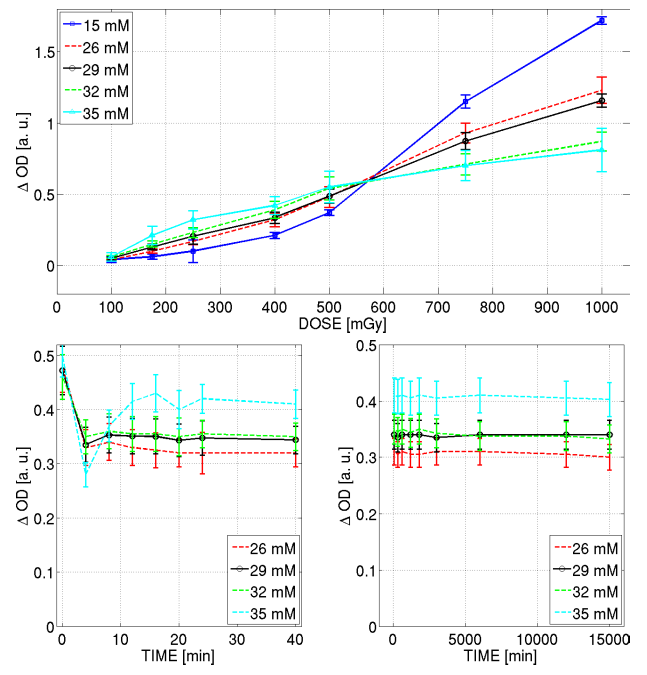


Figure 4.- Dose-response of FXG with different concentrations of sulfuric acid (top) and corresponding short/long term read out stability (bottom) delivering 750 mGy.

Therefore, after analyzing the linear trend of the sensitivity response as a function of increasing the amount of sulfuric acid in the solution (as evidenced in Figure 4), it was suitable to continue with further variations of component concentrations. The quantity of sulfuric acid was set to 29 mM, which is the representative midpoint. It was found that 29 mM sulfuric acid concentration presents the best post-irradiation stability when compared with the other concentrations, as reported in Figure 4. Although not here reported, similar behavior as reported in Figure 4 was observed for the range of sulfuric acid concentrations investigated (15-40 mM). After preliminary examination, the results obtained from varying the concentration of ferrous ammonium sulfate (FAS) practically showed increased sensitivity for greater amounts of FAS available to interact and react with the incident radiation, as shown in Figure 5. However, it was observed that saturation happens at lower doses when the amount of FAS is increased. As a consequence, although the sensitivity might be improved, the corresponding dose range of validity for the linearity would be significantly reduced.


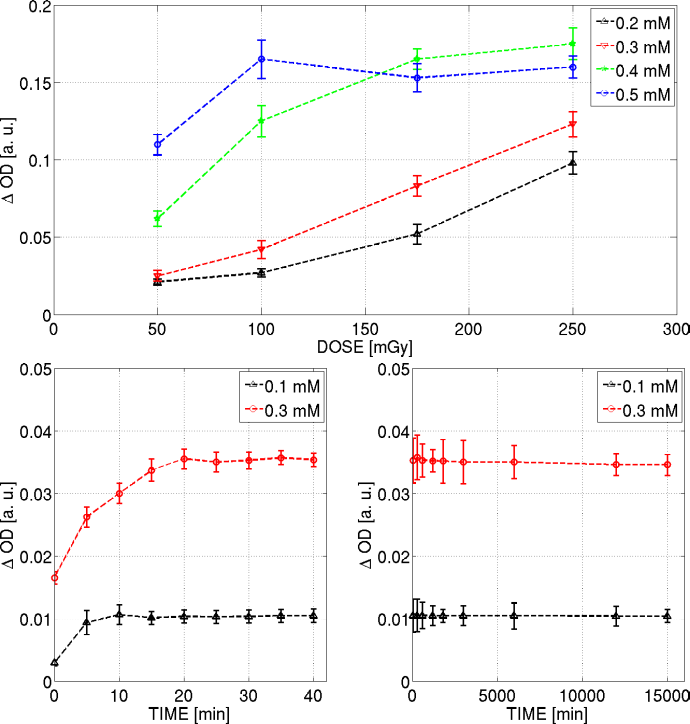


Figure 5.- Dose-response of FXG with different concentrations of FAS (top) and short/long term read out stability at different XO concentrations (bottom) delivering 750 and 250 mGy to the formulations, respectively.

The composition corresponding to 0.3 mM of FAS; keeping concentration of other components to: 29 mM sulfuric acid, 0.1 mM XO, 5 mM benzoic acid dissolved in a matrix consisting of 124.38 mM gelatin and 96% of water; in particular showed sufficient enough sensitivity to differentiate low dose values as 50 mGy and it was also verified that this formulation attains acceptable stability because the Δ*OD* mean value remained almost constant after 20 minutes. Conversely, the solution with 0.2 mM of ferrous ammonium sulfate was not able to differentiate dose values lower than 175 mGy. Finally, it was found that preparation with the concentration of 0.5 mM FAS did not exhibit linear response within an acceptably wide enough dose range. Therefore, the concentration of 0.3 mM of FAS was used to continue the elaboration process to improve sensitivity for low dose levels.

Regarding the variation of XO, the obtained results showed that this component is closely related to the stability of the system. Stability studies regarding elapsed time post-irradiation suggest that the composition prepared with 0.1 mM XO is more stable, as reported in Figure 5. Although both XO concentrations 0.1 mM and 0.3 mM exhibit acceptable stability after 20 minutes, it has to be mentioned that long term stability (10 hours) shows better performance for 0.1 mM concentration of XO, as reported in Figure 5.

Finally, the composition corresponding to the higher sensitivity for the purposes of this study consists of: 5 mM benzoic acid, 29 mM sulfuric acid, 0.3 mM ferrous ammonium sulfate and 0.1 mM XO. All components were suspended in a gel matrix gel 124.38 mM porcine skin gel (300 Bloom) to 96% of volume with tri-distilled water.

The output (dose-response curves) obtained through the change in optical density with respect to the absorbed dose showed a linear behavior with two well-defined slopes, as seen in Figure 6.It was possible to achieve high enough resolution for dose levels even lower than 20 mGy that were clearly distinguishable from background.


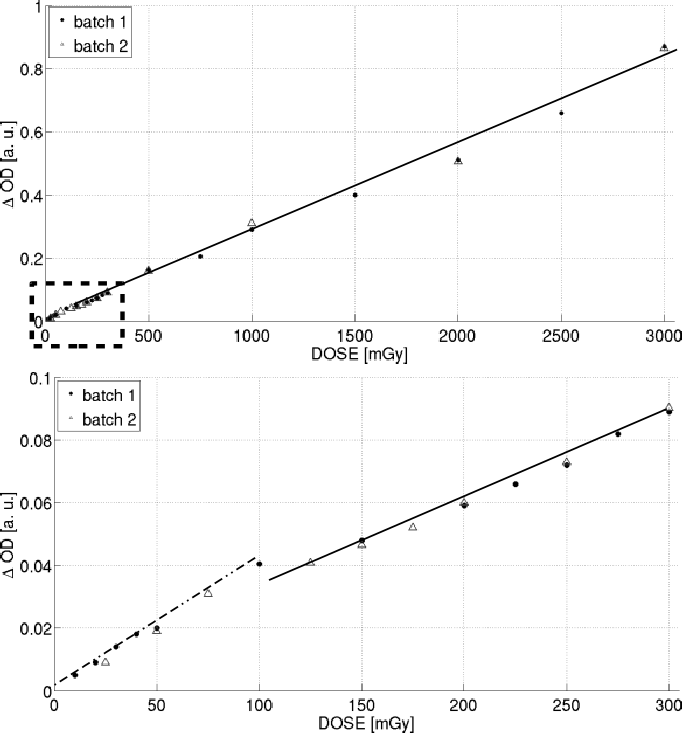


Figure 6.- Dose-response of FXG along with reproducibility for different elaboration batches (top) and zoom for the region indicated by rectangle (bottom). Linear regression parameters are: (4.12±0.08)×10^-4^ and R^2^=0.997 for the dose range 20-100 mGy and (3.79±0.15)×10^-4^ and R^2^=0.992 for the dose range 100-3000 mGy.

This behavior is due to changes in the nature of the solution due to the exhaustion of any of its components. In fact, the sensitivity tests indicate that the component that mainly affects this behavior is the ferrous ammonium sulfate. Incorporating processing protocols and process improvements achieved sufficient sensitivity to distinguish dose levels below 20 mGy, maintaining linear behavior with a double slope at a point 100 mGy and similar consistency for all preparations.

**3.2.- Characterization of the developed high sensitivity Fricke gel dosimeter**

Three different elaborations of the optimized composition were evaluated, changing absorbed dose ranges during irradiation. Sample positioning in phantom and further read out were maintained, hence ensuring that the obtained results could be further compared in order to investigate if the output remained stable regardless of the method of characterization.

Two different batches of high sensitivity Fricke solution were prepared separately. The first batch was evaluated for the range of 10-3000 mGy; whereas the second one was irradiated with doses in the interval 25-3000 mGy. As presented in Figure 6, the same behavior of linearity was observed between solutions regardless of characterization method.

Table 1 reports measurements of average absorbed doses corresponding to different beam qualities according to applied accelerating voltages.

Table 1.- Dose from dosimeter response (*Δ OD*) for different radiological beam qualities

| Accelerating voltage [kVp] | Reference delivered dose: 125 mGy | Reference delivered dose: 250 mGy |
| --- | --- | --- |
| 20 | 0.14±0.05 | 0.27±0.06 |
| 25 | 0.13±0.07 | 0.25±0.06 |
| 45 | 0.14±0.07 | 0.24±0.06 |
| 50 | 0.15±0.06 | 0.26±0.07 |

The results reported in Table 1 support that this dosimetry system response seems to be independent of beam quality, due to statistically indistinguishable results. The independence of beam quality could be also evaluated in terms of sensitivity of the system (characterized by the slope of linear regression) for the different accelerating voltages.

This preliminary evidence suggests that the developed sensitive dosimeter is practically independent of beam quality, as least in radiological applications.

**3.3.- Implementation of the dosimeter for radiology**

Tomography imaging of cylindrical phantom with inserted ionization chamber was performed in correspondence with Figure 7. The reconstructed images showed an internal structure of the ionization chamber clearly differentiated from the water surrounding it. Ionization chamber sensitive (ionization) volume consists mainly of air and the electronics made of metallic pieces that produce changes in the X-ray images when comparing ionization chamber and water. (The latter is conventionally considered as reference for human (soft) tissue; *i.e.* radiological equivalent to water.)


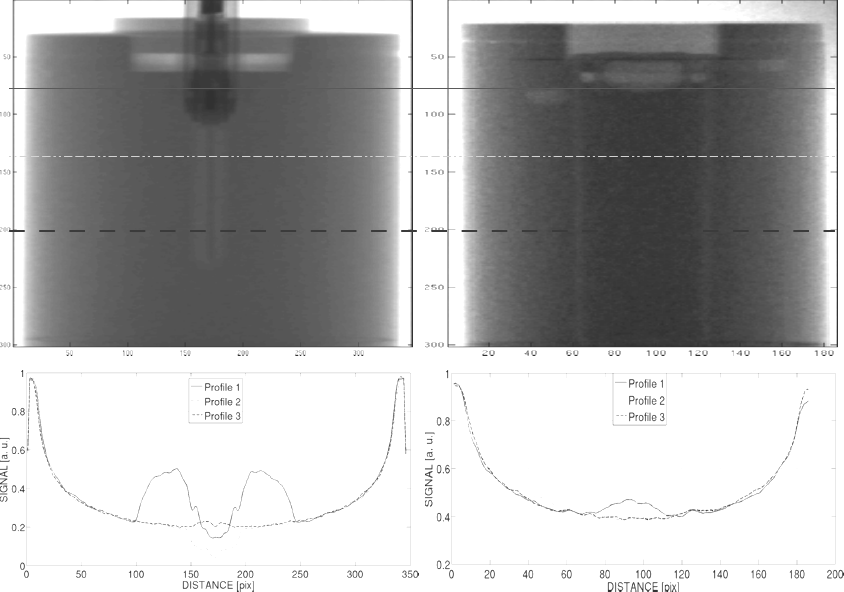


Figure 7.- Absorption contrast from µCT of phantom containing ionization chamber (top left) and high sensitivity FXG (top right) along with profiles at different locations (bottom).

Conversely, in the case of the corresponding tomographic projection images obtained from FXG dosimeter presented in Figure 7, it was confirmed that when placing the FXG dosimeter within a water equivalent medium, there are almost non-differences in acquired signal. This suggests very similar radiation absorption and scattering properties between water and dosimeter. It indicates, therefore, good water equivalence, thus meaning (soft) tissue equivalence for clinical applications, as mentioned.

The calibration curve was obtained for the solution implemented in this test, getting absorbance sensitivity of (48 ± 3) × 10^-4^/mGy and correlation coefficient R^2^ = 0.998, as shown in Figure 8. The obtained fit is valid and applies to the investigated dose range 25-200 mGy. Data reported in Figure 8 correspond to mean values and associated standard deviation obtained from 5 samples performing independent measurements for each dose value.


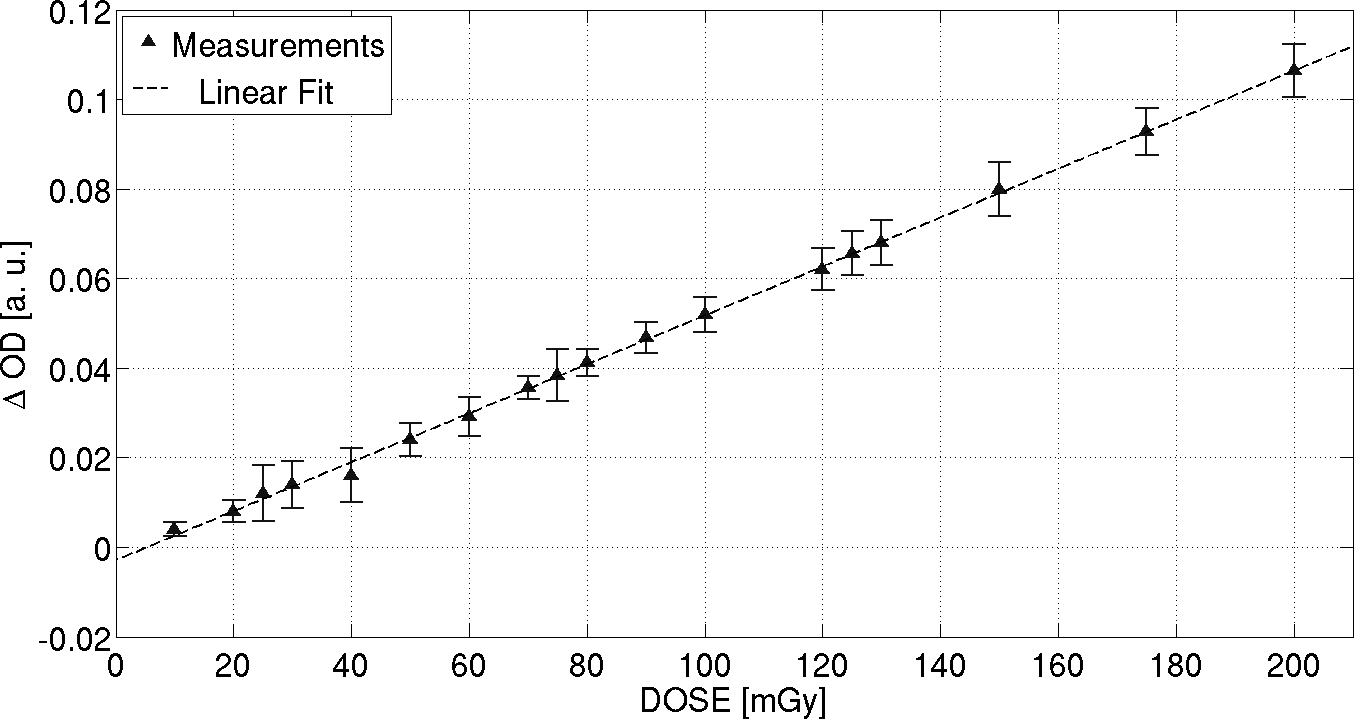


Figure 8.- Calibration curve of high sensitivity FXG dosimeters used for µCT applications.

For dosimetric characterization of µCT irradiation five vials were placed inside a water-equivalent phantom that was imaged in the µCT facility, as sketched in Figure 9.


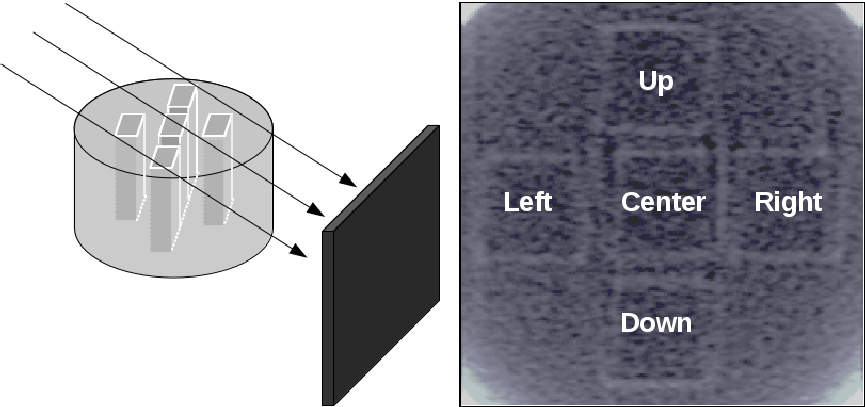


Figure 9.-Sketch of µCT setup showing X-ray beam, phantom with five FXG vials and detector (left) and reconstructed CT slice (right).

The object was imaged using a scanning modality of short acquisition times. The developed high sensitivity benzoic FXG dosimeters and ionization chamber were used to perform measurements during the µCT scanning process that lasted 12 minutes using an intensity-modulated beam. The obtained results are reported in Table 2.

Table 2.- Dose measurements (in mGy) during CT irradiation

| Dosimeter location (Figure 9) | Ion. Chamber | | High sensitivity Fricke gel |
| --- | --- | --- | --- |
| Center | | 134.1±0.1 | 118.0±3.1 |
| Up | | 78.6±0.1 | 74.5±8.2 |
| Down | | 100.6±0.2 | 94.2±6.4 |
| Left | | 123.7±0.2 | 121.1±4.5 |
| Right | | 100.0±0.1 | 102.0±2.1 |

The relative poor quality of the reconstructed slice, presented in Figure 9, is due to the selected scanning modality with short irradiation times and reduced quantity of angular projection acquisition. This issue needs to be carefully investigated when performing µCT scanning, because high quality imaging may require excessively large exposures thus avoiding its applications for *in-vivo* cases.

**4.- DISCUSSION**

First of all, it should be emphasized that the overall performance of the developed dosimetric system exhibits typical characteristics of gel dosimeters, like linear response within dose range of interest [Valente 2007 b, Schreiner 2004], reproducibility [Valente 2007 b, Gambarini 2006, Valente 2007], tissue-equivalence [Valente 2007, Gambarini 2006, Baldock 2006, Valente 2007, quite negligible photon beam energy dependence [Schreiner 2004, Baldock 2006, Gupta 1979, Sanguanmith 2011, Klassen 1999].

Regarding preparation of FXG sensitive material, the approach of testing output response in correspondence to different chemical compositions, varying one component at a time, allowed to suitably improve dose response sensitivity. Once a high enough sensitive composition was achieved, further studies regarding stability were conducted in order to extend the available parameters for helping in composition selection.

Keeping controlled thermal history is recommended in order to avoid auto-oxidation. The criterion of fixing storage time to 24 hours for the Fricke solution with benzoic acid was established to this aim. The effect of storage time on dose response was previously checked; authors observed that longer storage times impact gel color. Moreover, excessively long elapsed time between gel elaboration and irradiation resulted in darker dosimeters (noticed with naked eye), thereby decreasing corresponding sensitivity. Before proceeding with experiments, the elapsed time between preparation and utilization that is required to arrive at chemical stability was carefully investigated. Also, Fricke solution preparation was always limited to batches not exceeding 500 ml filling standard vials (4 ml). Therefore, no large gel volumes were prepared for this work in order to avoid complications associated with long storage periods for reaching steady state conditions. In this sense, it should be addressed that caution is needed when analyzing whether results obtained with cuvettes might be applicable to dosimeters of larger volumes.

The dedicated optical analysis technique for the characterization of the high sensitivity FXG dosimeter demonstrated remarkable capability for assessing absorbed dose by means of transmission/absorption measurements. However, the implemented methodology, which appeared as optimal for analyzing homogeneous dose distribution within the sample, would need to be suitably modified if non-uniform dose distribution needed to be measured. In fact, one of the characteristics of the analyzing method that might be considered *a priori* as a limitation is that recorded dose values correspond to the integral along the optical path, thus providing an estimation of an “effective” dose value that obviously is not able to distinguish different doses along the integration path. That is why FXG samples should be prepared in “small enough” containers so that, when irradiated, it might be reasonable to consider that absorbed dose is constant within sample path.

Nevertheless, this potential drawback is suitably overcome by an improved 2D analyzing technique based on almost monochromatic sample transmission imaging [Gambarini 2004, Gambarini 2006, Carrara 2011, Gambarini 2002, Gambarini 2007, Valente 2007].

As expected, the presence of linearity in dose-response is reduced to specific dose ranges, observing initial dose interval without appreciable response as well as saturation effects at higher dose levels. Moreover, the extension of this linearity range shows to be strongly correlated with linear slope, which represents the sensitivity of the system. The behavior at doses lower than linearity range corresponds to threshold effects exhibiting *supra* linear trend; whereas for doses higher than linearity range the output presents typical saturation effects, then *sub* linear trend, due to extinction of available reactive components.

It is expected that both sulfuric and benzoic acids concentrations should significantly influence the output, because the change in medium pH highly influences overall reactive grade. Similarly, concentration of FAS being the component providing the reactive agents (ferrous oxide) affects output. These results are in agreement with previous studies [Svensson 1979, Valente 2007, Molina 2013*,* Dewhurst 1951].

Sulfuric acid concentration needs to be carefully investigated. Its significant influence of dose-response in hydrogels for dosimetry has been well reported [Svensson 1979, Dewhurst 1951, Palm 2000]. The amount of sulfuric acid used in this work was previously studied [Molina 2013] in order to avoid excessive reactivity but the highest sensitivity possible was maintained. Additionally, sulfuric acid concentration was conditioned to ensure acceptable post-irradiation stability, thus allowing flexibility for read out processes. As mentioned in previous works, it is clear that sulfuric acid used for the preparation of the Fricke dosimeter solution may contain trace impurities that can affect the yield of ferric ions. Small amounts of impurities, mainly organic, can affect the yield of ferric ions G(Fe^3+^). This issue is not accounted for during FXG preparation whenever the objective is limited to relative dosimetry, but it should be emphasized that it is required for absolute dosimetry. Regardless, it was not the intent of this study to exactly identify the cause of the effect and interested readers may refer to literature pertaining to this topic [Healy 2004, Gupta 1985 b]. Thus, it was enough to assess adequate amount of sulfuric acid that satisfied sensitivity, reactivity and stability requirements.

The effect of XO on dose-response was carefully investigated during preliminary studies before the selection of adequate composition. Many authors reported that smaller changes in dose-response correspond to changes in XO concentrations of 0.1 mM [Gupta 1973, Svensson 1979, Molina 2013, Dewhurst 1951, Palm 2000]. The same was found with the preliminary results obtained for selecting dosimeter composition for this work. However, it was shown that different XO compositions, as may happen when using different suppliers -or even different lots from the same manufacturers- significantly affect dose-responses [Healy 2004]. It was reported, in fact, that XO dye contains impurities, like cresol (red), acids (iminodiacetic for example) and other not pure but semi-XO compounds [Molina 2013]. Moreover, different XO compositions from different manufacturing processes may impact the chemical yield *G* value [Gupta 1983, Gupta 1970, Murakami 1967, Babic 2008]. Preliminary tests for this work were conducted in order to characterize the performance of XO from different suppliers and lots (Anedra U.S. lot 13445-11 and two flasks from Sigma-Aldrich lots 52099A). After examination, Sigma-Aldrich XO was selected, because it exhibits higher sensitivity. Clearly, higher sensitivity is particularly critical when looking for low dose level dosimetry. Therefore, preliminary studies to establish the most convenient XO are a critical issue when looking for improved performance.

Regarding the performance of the dosimetric system for radiological applications, Figure 7 presents dose-response for the specific batch used for further measurements, which is needed for conversion to dose vials, (used to measure the absorbed dose during X-ray µCT scanning). In order to anticipate dose range, measurements with ionization chamber were performed before. This way it was possible to determine, *a priori*, that absorbed dose value should be lower than 100/120 mGy. Hence, the selected range (below 200 mGy) reported in Figure 8 for obtaining calibration curve seemed to be a good choice. Values reported in Figure 8 correspond to the average of 5 samples for each dose level. The selection of the optimal chemical composition for low dose level dosimetry was based on the compromise between high sensitivity (high slope values), extension of linearity range and composition output stability, both after preparation and after irradiation. The obtained results for measuring low level absorbed dose in CT-like set up, some of them summarized in Table 2, evidence that the developed dosimetry system is capable of assessing similar dose estimations to ionization chamber, here used as an alternative for conventional systems.

A critical issue must be emphasized at this time: calibration curve provides information for dose conversion if necessary, but it samples for calibration must come from the same elaboration batch. The point in question is the importance of performing specific calibration for each FXG elaboration batch. For example, Figure 8 reports dose-response calibration for the specific batch that was further used for X-ray µCT measurements, thus showing that each batch needs its own calibration. Moreover, it would be helpful to develop processes for batch-to-batch cross-comparisons, which might be carefully implemented, attempting to integrate results from different elaboration batches.

Despite the noticeable overall agreement between high sensitivity FXG dosimeter and conventional method, ionization chamber and FXG dosimeter measurement volumes do not agree. Therefore it should be expected that different dose readout values would be recorded if non-uniform irradiation is performed. Furthermore, as pointed out by results reported in Figure 5, the presence of non-water equivalent components of the ionization chamber might affect, even in a minor but potentially non-negligible way, the radiation field, thus raising non-desired measurement alterations.

Regarding the dose-response calibration of the batch dedicated to characterization of µCT facility, it must be pointed out that characterization of linear trend for low doses require further extensive investigations to get definitive characterization in terms of the different internal chemical processes affecting dose-response curve. Therefore, dose was measured with ionization chamber in the same conditions as gel samples, thus attempting to get the most reliable comparison. The presence of dose gradients during µCT scanning cannot be avoided and there is no absolute guarantee that the dose gradients do in fact affect ionization chamber and gel dosimeters in an identical manner.

**5.- CONCLUSIONS**

The purpose of developing, characterizing and preliminarily implementing a tissue-equivalent high sensitivity FXG dosimeter was accomplished satisfactorily. It was possible to improve the sensitivity of the system, characterized by the slope in the dose-response curve, obtaining a formulation 50 times, approximately, more sensitive than the standard one (see Figure 3). Besides, it is important to underline that the present study does not intend to establish that benzoic acid would be indispensable for developing FXG with higher sensitivity.

It was demonstrated that novel FXG dosimetry system designed for low dose ranges certainly exhibits linear response being capable of reliably measuring dose values within the range of 25-3000 mGy, observing readouts clearly distinguishable from the background and uncertainties less than 7% in all cases. Comparisons of gel dosimeters with ionization chamber suggest promising performance of the developed dosimeter for low dose levels.

The developed dosimetry system exhibits similarity to conventional system in the dose record, being indeed an indication that the developed system might constitute a promising tool as radiation detector potentially applicable to dosimetry in radiology and radiotherapy practices. The developed benzoic FXG was capable to perform reliable measurements of absorbed dose during µCT scanning. Although these measurements are preliminary carried out in water-equivalent phantoms, the information collected is useful to predict the range of doses absorbed during µCT scanning of small animals. Next step involves the use of dedicated phantoms constructed according to animal morphology to insert benzoic Fricke gel dosimeters avoiding the use of ionization chamber and associated limitations due to movements during µCT scanning.

**Acknowledgments**

This work was partially supported by project “Innovation Systems Research for Radiation Dosimetry: phase I (**ISIDORA I**) and phase II (**ISIDORA II**)” funded by University of Cordoba, Argentina and project **ESPORA I** (PIP 2014-2016 11220130100658CO) funded by CONICET, Argentina.

**REFERENCES**

1. [Schreiner 2004] Schreiner L. *Review of Fricke gel dosimeters*. Jour. Phys. Conf. Ser. **3**, pp. 9-21, 2004.
2. [Baldock 2006] Baldock C. *Historical overview of the development of gel dosimetry: a personal perspective*. Jour. Phys. Conf. Ser. **56** pp. 14-22, 2006.
3. [Valente 2007] Valente M. *Fricke gel dosimetry for 3D imaging of absorbed dose in radiotherapy.* PhD. Dissertation. University of Milan. Italy, 2007.
4. [Svensson 1979] Svensson H. and Brahme A. *Ferrous sulphate dosimetry for electrons.* Acta Radiol. Oncol. **18** pp. 326-336, 1979.
5. [Sanguanmith 2011] Sanguanmith S.; Muroya Y.; Tippayamontri T.; Meesungnoen J.; Lin M.; Katsumura Y. and Jay-Gerin J. *Temperature dependence of the Fricke dosimeter and spur expansion time in the low-LET high-temperature radiolysis of water up to 350 °C: a Monte-Carlo simulation study.* Phys. Chem. Chem. Phys. **22** pp. 10690-10698, 2011.
6. [Valente 2007b] Valente M.; Gambarini G.; Bartesaghi; Gambarini G.; Brusa D.; Castellano G. and Carrara M. *Fricke gel Dosimeter Tissue Equivalence: a Monte Carlo Study.* Proccedings of the 10^th^ International Congress on Astroparticles, Particles and Space Physics, Detectors and Medical Physics and Applications.Villa Olmo, Italy pp. 605-609, 2007.
7. [Valente 2012] Valente M.; Molina W.; Tirao G.; Graña D. and Perez P. *Methods for Novel tissue-equivalent chemical dosimeter optimized for radiological low dose levels.* Proc. 11^th^ International Low Radiation Conference. Lyon, France, 2012.
8. [Klassen 1999] Klassen N.; Shortt K.; Seuntjens J. and Ross C. *Temperature Fricke dosimetry: the difference between G(Fe3+) for 60Co gamma-rays and high-energy x-rays.* Phys. Med. Biol. **44** pp. 1609-1624, 1999.
9. [Gupta 1975] Gupta B. and Gomathy K. *Consistency of Ferrous Sulphate-Benzoic Acid-Xylenol Orange dosimeter*. Int. Jour. Appl. Rad. Isot. **25**, pp. 509-513, 1975.
10. [Vedelago 2014] Vedelago J.; Quiroga A. snd Valente M. *Characterization of ferric ions diffusion in Fricke gel dosimeters by using inverse problem techniques.* Jour. Rad. Eff. Def. Solids **169** (10) pp. 845-854, 2014.
11. [Gambarini 2006] Gambarini G.; Brusa D.; Carrara M.; Castellano G.; Mariani M.; Tomatis S.; Valente M. and Vanossi E. *Dose Imaging in radiotherapy photon fields with Fricke and Normoxic-polymer Gels.* Nucl. Jour. Phys. Conf. Ser. **41**, pp. 466-474, 2006.
12. [Carrara 2007] Carrara M.; Gambarini G.; Tomatis S. and Valente M. (2007). *Dose distribution measurements by means of gel-layer dosimeters.evaluation of algorithms for artefacts amendment.*Nucl. Instr. Rad. Res. A **579**: 334-338, 2007.
13. [Molina 2013] Molina W. *Development and caracterization of a Fricke gel system for dosimetry in radiology.* MSc. Dissertation. Institute of Scientific Investigations of Venezuela, 2013.
14. [Gupta 1998] Gupta B. and Nilekani S. *Ferrous ion oxidations by H, OH and H_2_O_2_ in aerated FBX dosimetry system model and instrumentation development for in-situ Fricke gel dosimetry in clinical environment.* Radiation Physics and Chemistry **53** pp. 643-650, 1998.
15. [Gupta 1985] Gupta B.; Bhat R.; Narayan D. and Nikelani S. (1985). *Dose-response A spectrophotometric method for free radical dosimetey.* Radiat. Phys. Chem. ***26*** *pp. 647-656,* 1985.
16. [Gupta 1973] Gupta B. *Microdetermination Techniques for H_2_0_2_ in Irradiated Solutions*. Microchem. Jour. **18**, pp. 363-374, 1973.
17. [Gambarini 1994] Gambarini G. and Arrigoni S. *Dose-response curve slope improvement and result reproducibility of ferrous sulphate-doped gels analysed by NMR imaging.* Phys. Med. Biol ***39*** *pp. 703-717, 1994.*
18. [Gambarini 2004] Gambarini G.; Birattari C.; Mariani M.; Marchesini R.; Pirola L.; Prestini P.; Sella M. and Tomatis S. *Study of light transmittance from layers of Fricke-xylenol-orange-gel dosimeters.* Nucl. Instr. Rad. Res. B **213**, pp. 321-324, 2004.
19. [Tomatis 2007] Tomatis S.; Carrara M.; Gambarini G.; Marchesini R. and Valente M. *Gel-layer dosimetry for dose verification in intensity modulated radiation therapy.* Nucl. Instr. Rad. Res. A **579**, pp. 506-509, 2007.
20. [Valente 2007c] Valente M.; Aon E.; Brunetto M.; Castellano G.; Gallivanone F. and Gambarini G. *Gel dosimetry measurements and Monte Carlo modelling for external radiotherapy photon beams. Comparison with a treatment planning system.* Nucl. Instr. Rad. Res. A **579** pp. 497-501, 2007.
21. [Castellano 2007] Castellano G.; Brusa D.; Carrara M.; Gambarini G. and Valente M. *An optimized Monte Carlo (PENELOPE) code for the characterization of gel-layer detectors in radiotherapy.* Nucl. Instr. Rad. Res. A **579** pp. 502-505, 2007.
22. [Carrara 2011] Carrara M.; Gambarini G.; Borroni M.; Cerrotta A.; Fallai C.; Invernizzi M.; Cavatorta C. and Zonca G.. *Fricke gel dosimetric catheters in high dose rate brachytherapy. In phantom dose distribution measurements of a 5 catheter implant. Rad. Meas*. **46** (12) pp. 722-725, 2011.
23. [Gambarini 2010] Gambarini G.; Bartesaghi G.; Burian J.; Carrara M.; Marek M.; Negri A.; Pirola L. and Viererbl L. *Fast-neutron dose evaluation in BNCT with Fricke gel layer detectors. Rad. Meas.* **45** (10) pp. 1398-1401, 2010.
24. [Gambarini 2002] Gambarini G.; Agosteo A.; Marchesi P.; Nava E.; Palazzi P.; Pecci A.; Rosi G. and Tinti R. *Discrimination of various contributions to the absorbed dose in BNCT: Fricke-gel imaging and intercomparison with other experimental results.* Rad. Prot. Dosim. **101**, pp. 419-422, 2002.
25. [Gambarini 2004 b] Gambarini G.; Colli V.; Gaya S.; Petrovich C.; Pirola L. and Rosi G. *In-phantom imaging of all dose components in boron neutron capture therapy by means of gel dosimeters.* App. Rad. Isot. **61** pp. 759-763, 2004.
26. [Gambarini 1997] Gambarini G.; Monti D.; Fumagalli M.; Birattari C. and Salvadori P. *Phantom Dosimeters Examined by NMR Analysis: a Promising Technique for 3-D Determination of Absorbed Dose.* App. Rad. Isot. **48** pp. 1477-1484, 1997.
27. [Bartesaghi 2009] Bartesaghi G.; Burian J.; Gambarini G.; Marek M.; Negri A. and Viererbl L. *Evaluation of all dose components in the LVR-15 reactor epithermal neutron beam using Fricke gel dosimeter layers.* App. Rad. Isot. **67** pp. 199-201, 2009.
28. [Gambarini 2007] Gambarini G.; Moss R.; Mariani M.; Carrara M.; Daquino G.; Nievaart V. and Valente M. *Gelmdosimeters as useful dose and thermal-fluence detectors in boron neutron capture (BNCT).* Jour. Rad. Ef. Deff. Solids **162** pp. 777-783, 2007.
29. [Gambarini 2014] Gambarini G.; Negri A.; Regazzoni V.; Magni D.; Nolli R.; Campi F.; Burian G.; Marek M.; Klupak V. and Viererbl L. *Methods for dose measurements in small phantoms irradiated at BNCT epithermal column.* App. Rad. Isot. In press, 2014.
30. [Gupta 1982] Gupta B.; Kini U.; Bhat, R. and Madhvanath, U. *Use of the FBX dosemeter for the calibration of cobalt-60 and high-energy teletherapy machines*. Phys. Med. Biol. **27** pp. 235-245, 1982.
31. [Wnder 1968] Wander R.; Neta P. and Dorfman L. *Pulse radiolysis studies. XII. Kinetics and spectra of the cyclohexadienyl radicals in aqueous benzoic acid solution*. J. Phys. Chem. **72**, pp. 2946-2949, 1968.
32. [Gore 1984] Gore J.; Kang Y. and Schulz R. *Measurement of radiation dose distribution by nuclear magnetic resonance (NMR) imaging*. Phys. Med. Biol. **29** pp. 1189-1197, 1984.
33. [Kelly 1998] Kelly R.; Jordan K. and Battista J. *Optical CT reconstruction of 3D dose distributions using the ferrous-benzoic-xylenol (FBX) gel dosimeter*. Med. Phys. **25** pp. 1741-1750, 1998.
34. [Gupta 1973b] Gupta B. *Low-level dosimetric studies with FeSO^-^_4_ benzoic acid-xylenol orange system*. Proc. Dosim. Agriculture, Industry, Biology and Medicine IAEA, pp. 421-432, 1973.
35. [Upadhyay 1982] Upadhyay S.; Reddy A.; Gupta M. and Nagaratnam A. A Tissue-Equivalent Modified FBX Dosimetric System. Int. J. Appl. Radiat. Isot. **33**, pp. 47-49, 1982.
36. [Olding 2011] Olding T. and Schreiner J. *Cone-beam optical computed tomography for gel dosimetry II: imaging protocols*. Phys. Med. Biol. **56** pp. 1259-1279, 2011.
37. [Dewhurst 1951] Dewhurst H. Letter: *Effect of organic substances on the γ -ray oxidation of ferrous sulfate.* J. Chem. Phys. **19**, 1951.
38. [Palm 2000] Palm A. and Mattsson, O. *Influence of sulphuric acid contaminants on Fricke dosimetry* Phys. Med. Biol. **45**, pp. N111-N114, 2000.
39. [Healy 2004] Healy B.; Brindha S.; Zahmatkesh M. and Baldock C. *Characterisation of the ferrous-xylenol orange-gelatin (FXG) gel dosimeter*. Jour. Phys. Conf. Ser. **3** pp. 142-145, 2004.
40. [Gupta 1985b] Gupta B. and Narayan G. *G(Fe3+) values in the FBX dosemeter*. Phys. Med. Biol. **30**(4) pp. 337-340, 1985.
41. [Gupta 1983] Gupta B.; Bhat M.; Narayan G. and Susheela B. *Acid and xylenol orange effects in the FBX dosimeter*. Int. Jour.Ap. Rad. Isot. **34** (6), pp. 887-890, 1983.
42. [Gupta 1970] Gupta B. *Radiolisis of ferrous-xylenol orange system in acid solutions*. Proc. Symposium on Radiation Chemistry, Bombay India pp. 115-125, 1970.
43. [Murakami 1967] Murakami M.; Yoshino T. and Harasawa S. *Separation and acid equilibria of xylenol orange and semi-xylenol orange*. Talanta **14**(11) pp. 1293-1307, 1967.
44. [Babic 2008] Babic S.; Battista J. and Jordan, K. *An apparent threshold dose response in ferrous xylenol-orange gel dosimeters when scanned with a yellow light source*. Phys. Med. Biol. **53** pp. 1637-1650, 2008.
